# Sectoral activation of glia in an inducible mouse model of autosomal dominant retinitis pigmentosa

**DOI:** 10.1038/s41598-020-73749-y

**Published:** 2020-10-12

**Authors:** Michael T. Massengill, Neil F. Ash, Brianna M. Young, Cristhian J. Ildefonso, Alfred S. Lewin

**Affiliations:** 1 Department of Molecular Genetics and Microbiology, University of Florida College of Medicine, Gainesville, FL 32601 USA; 2 Department of Ophthalmology, University of Florida College of Medicine, Gainesville, FL 32601 USA

**Keywords:** Retinal diseases, Glial biology

## Abstract

Retinitis pigmentosa (RP) is a group of blinding disorders caused by diverse mutations, including in rhodopsin (*RHO*). Effective therapies have yet to be discovered. The I307N *Rho* mouse is a light-inducible model of autosomal dominant RP. Our purpose was to describe the glial response in this mouse model to educate future experimentation. I307N *Rho* mice were exposed to 20,000 lx of light for thirty minutes to induce retinal degeneration. Immunofluorescence staining of cross-sections and flat-mounts was performed to visualize the response of microglia and Müller glia. Histology was correlated with spectral-domain optical coherence tomography imaging (SD-OCT). Microglia dendrites extended between photoreceptors within two hours of induction, withdrew their dendrites between twelve hours and one day, appeared ameboid by three days, and assumed a ramified morphology by one month. Glial activation was more robust in the inferior retina and modulated across the boundary of light damage. SD-OCT hyper-reflectivity overlapped with activated microglia. Finally, microglia transiently adhered to the RPE before which RPE cells appeared dysmorphic. Our data demonstrate the spatial and temporal pattern of glial activation in the I307N *Rho* mouse, and correlate these patterns with SD-OCT images, assisting in interpretation of SD-OCT images in preclinical models and in human RP.

## Introduction

Retinitis pigmentosa (RP) is a group of monogenic blinding disorders characterized by a primary phase of rod photoreceptor apoptosis followed by cone photoreceptor death. The clinical effects of these mutations and the resulting pattern of photoreceptor loss are progressive, concentric loss of peripheral vision, and night vision, with complete blindness in advanced disease^1^. Mutations in rhodopsin (*RHO*) represent approximately twelve-percent of all RP cases and are usually inherited in an autosomal dominant pattern^2^. Over 200 mutations in *RHO* have been described to date^3^. RP remains uncurable and one barrier to the generation of therapeutic interventions is the paucity of animal models for preclinical efficacy studies since a variety of mechanisms lead to the diseased phenotype^4,5^.

The I307N *Rho* mouse model of light-induced adRP recapitulates some crucial aspects of patients with the B1 phenotype, including sectoral and variably penetrant retinal degeneration^6–9^. These characteristics lend to the use of the I307N *Rho* mouse in preclinical efficacy studies or mechanistic studies. Furthermore, the light-inducible and tunable nature of retinal degeneration in the I307N *Rho* mouse creates an opportunity for controlled experimentation when compared to other mouse models of RP that experience early retinal degeneration that may overlap with the final stages of retinal development in the postnatal period^7,10^.

The generation and characterization of the I307N *Rho* mouse model was conducted over a decade ago by Budzynski et al. and has gained popularity among research groups in recent years^6–8,10,11^. Gargini et al. were the first to report activation of microglia and Müller glia at an early time point following illumination of the I307N *Rho* mouse and suggested that morphologically distinct cells exist in the area of injury when compared to nearby, relatively unaffected retina. They proposed that the model could be an excellent resource for studying cone death, retinal inflammation, and inner retinal remodeling^8,10^. We recently characterized the time-course of light-induced retinal degeneration in this model with spectral-domain optical coherence tomography (SD-OCT). We demonstrated that a hyper-reflective signal and retinal swelling were evident in the acute period following light challenge, which was followed by near-complete loss of the outer nuclear layer (ONL) within a week. We argued that these SD-OCT findings could be utilized as endpoints to produce an optimized preclinical efficacy study^7^. Recently, Zhang et al. demonstrated that whole-body exercise in the I307N *Rho* mouse mitigated both retinal inflammation, characterized by reduced microglia adherence to the RPE, and visual decline^11^.

A large body of literature suggests that the balance of microglia and Müller glia activity and resultant inflammatory signaling during retinal degeneration helps determine the extent of photoreceptor death or survival^12,13^. Many unique therapeutic approaches that involve modulating the inflammatory response have been studied in the context of retinal degeneration in RP^14–16^. Despite clear evidence for microglial activation in the I307N *Rho* mice with the work of Gargini et al. and Zhang et al.^8,11^, the temporal and spatial pattern of activation of microglia and Müller glia in the I307N *Rho* mouse remains unexplored. Without understanding the natural history of glial activation in the I307N *Rho*  mouse, our ability to utilize this model for mechanistic studies or preclinical testing of anti-inflammatory interventions remains suboptimal.

To address this gap in knowledge, we utilized histologic techniques to demonstrate that microglia undergo rapid migration into the outer retina, expansion in number after CD45-positive monocytic cell infiltration, and morphological activation to an ameboid phenotype within three days of light exposure, before returning to a ramified configuration after ONL clearance. Müller glia exhibited a dramatically increased expression of glial fibrillary-acidic protein (GFAP) during retinal degeneration. Because we employed a method of overhead illumination designed to recapitulate the sector RP observed in patients with class B1 mutations^17^, these glial responses were sectoral, as more profound reactivity occurred in the inferior, damaged retina when compared to the relatively preserved superior retina. Interestingly, the hyper-reflective signal on SD-OCT demarcated morphologically activated microglia from ramified microglia on a co-isolated retinal flat-mount. During this analysis, we were surprised to discover rapid shifts in total retinal thickness (TRT), as the retina thinned during the first two hours after exposure to light then swelled beginning at four hours. Finally, migration and adherence of microglia to the RPE was a relatively late and transient event that occurred approximately one to two weeks after light exposure. Together, these results should help future studies to designate optimal time points for analysis of retinal glial cells and correlating the glial response with SD-OCT findings in animal models of RP.

## Results

### Retinal glial cells respond to damage in a sectoral pattern within the first week after light damage

**Figure 1:**
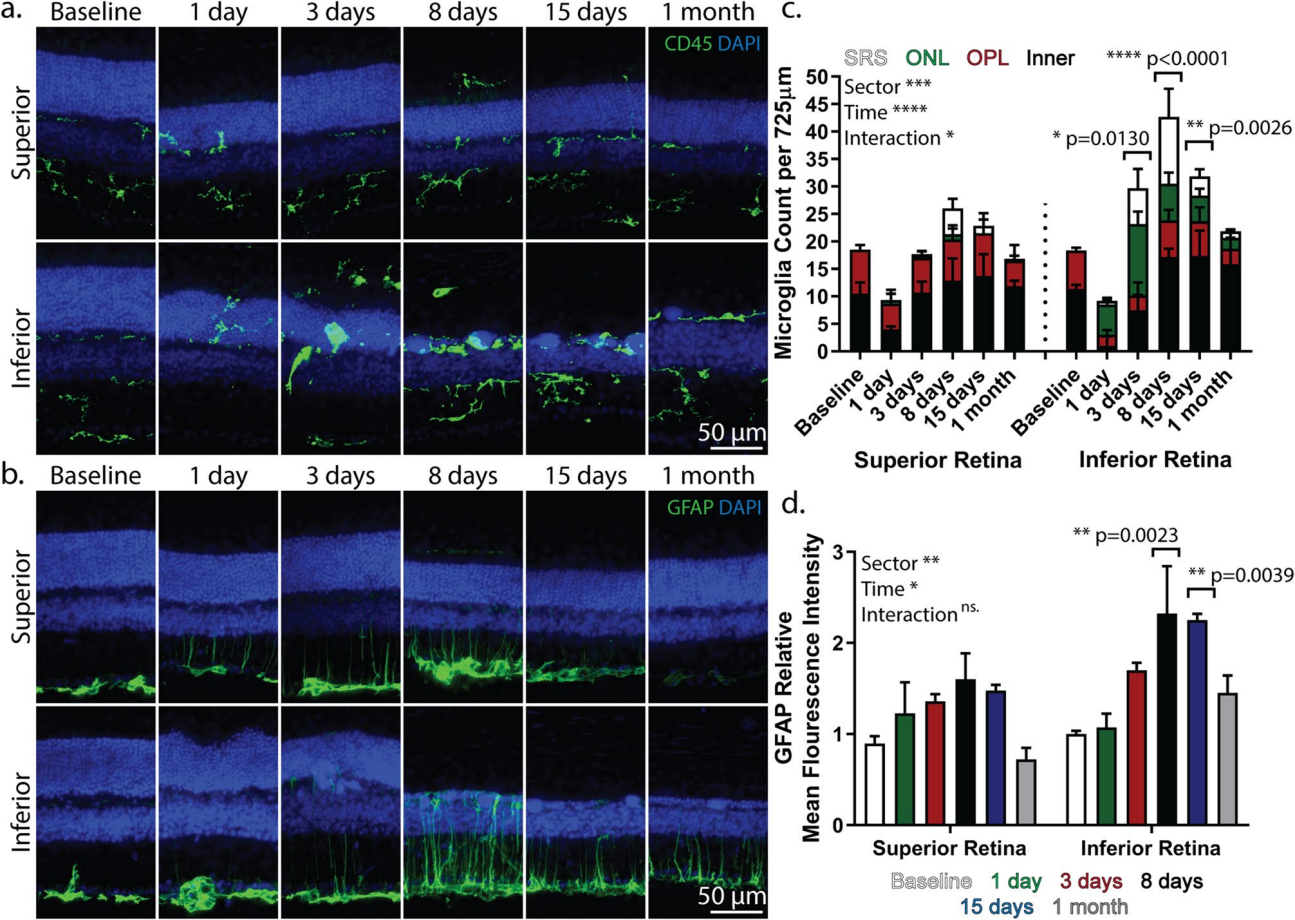
Activation of glial cells occurs in a sectoral pattern in the induced I307N *Rho* mouse with a more robust response in the inferior retina than superior retina. I307N *Rho* mice were exposed to thirty minutes of 20,000 lx of light and their eyes were extracted one day, three days, eight days, fifteen days or one month thereafter for IF-staining of PFA-fixed frozen sections. Sections were stained with (**a**) CD45 (green) and DAPI (blue) or (**b**) GFAP (green) and DAPI (blue). Images with an original magnification of 40 × were captured in the superior and inferior retina. (**c**) The number of microglia that appeared in the subretinal space (SRS), ONL, OPL, and inner retina (INL, IPL and GCL) were counted in two 40 × images (725 μm of length × 16 μm of thickness) in the inferior or superior retina per mouse at each time point and summed. Counts were performed by two masked observers then averaged (R^2^ = 0.8286). Bar graphs represent the mean count ± *s.e.m.* (*n* = *3* per time point). (**d**) GFAP expression was measured as the mean fluorescence intensity (MFI) within a polygon encompassing the area between the NFL and photoreceptor inner segments using ImageJ. The relative MFI was calculated as the MFI for a given image of the retina divided by the average MFI associated with the images of the inferior retina at baseline. Bar graphs represent the mean relative MFI ± s.e.m. (*n* = *3* per time point). Two-way repeated-measures ANOVA with matching across the retinal sector (mixed-model ANOVA) followed by Sidak’s multiple comparisons tests was performed to compare the total number of microglia or GFAP MFI at each time point to its corresponding within sector baseline. Normality was tested with the Shapiro–Wilk test of residuals. Of note, statistics were performed on raw MFI data. ns. = not significant; *p < 0.05; **p < 0.01; ***p < 0.001; ****p < 0.0001. Statistical data can be viewed in Supplementary Table S1 and S2.

We previously showed that exposure of the awake I307N *Rho* mouse to overhead, bright white-light results in inferonasal retinal degeneration^7^. Our rationale for employing overhead lighting rather than diffuse lighting via mirrored cages, which has been used elsewhere^6,8,10^, was to recapitulate the sectoral retinal injury seen in class B1 RP patients^9^. Gargini et al. have demonstrated that microglia progressed to an ameboid phenotype by day two after light exposure and that their activation occurred with regional dependence^8^. We thus expanded their analysis to determine whether microglia and Müller glia would follow a sectoral pattern of activation at other time points. To this end, we prepared frozen paraformaldehyde (PFA)-fixed cross-sections of the retina from I307N *Rho* mice before and one day through one month after light exposure. These sections were prepared along the superior-inferior axis to capture areas in the inferior retina that likely received the brunt of light exposure. Sections were subsequently stained with an antibody against CD45 (Fig. 1a), a receptor protein tyrosine phosphatase expressed in all nucleated hematopoietic cells^18^, or against GFAP, an intermediate filament associated protein that is expressed with Müller cell reactivity and in astrocytes^19^ (Fig. 1b).

The total number of CD45-positive cells were counted in the major retinal lamina of either the inferior retina or the superior retina (Fig. 1c). Importantly, CD45 staining co-localized with the ionized calcium-binding adapter molecule 1, an accepted marker of microglia^20^ (see Supplementary Fig. 1a). We chose to utilize the CD45 marker since it yielded more consistent results. Microglia showed a sectoral pattern of activation, as two-way repeat-measures ANOVA demonstrated that retinal sector is a significant independent factor and the total number of microglia was significantly higher only in the inferior retina, but not superior retina, compared to baseline during post-hoc testing. Furthermore, there were dramatic shifts in microglia localization within the retina. With the majority of ONL already degenerated by day eight, the microglial response appeared to resolve towards the baseline distribution. Microglia did not migrate to the ONL or subretinal space (SRS) in wild-type littermates exposed to the same light damage protocol one day prior (see Supplementary Fig. 1b).

To quantify the differences in GFAP-positivity in the inferior versus superior retina, we measured the mean fluorescence intensity (MFI) of each image relative to baseline (Fig. 1d). The statistical peak of GFAP accumulation occurred in the inferior retina on days eight and fifteen after illumination. Furthermore, the independent variable of retinal sector showed significance in two-way repeat-measures ANOVA, again suggesting regional differences in glial activation. Wild-type animals exposed to the light damage protocol did not develop GFAP-positive Müller cell processes (see Supplementary Fig. 1b). These results indicate that retinal degeneration in the I307N *Rho* mice induced both sectoral and temporal activation of microglia and Müller glia.

### Microglia undergo rapid and dramatic morphological changes and expand in number after monocyte infiltration

Next, we sought to improve our understanding of microglia in the acute phase after light challenge. I307N *Rho* mice were exposed to light and PFA-fixed retinal flat-mounts were prepared from early time points (e.g., within hours of the cessation of illumination) and up to one month thereafter. Flat-mounts were then stained with anti-CD45, anti-GFAP, anti-Iba1 and/or isolectin B4 (IB4) and CD45-positive cell counts were performed.

**Figure 2:**
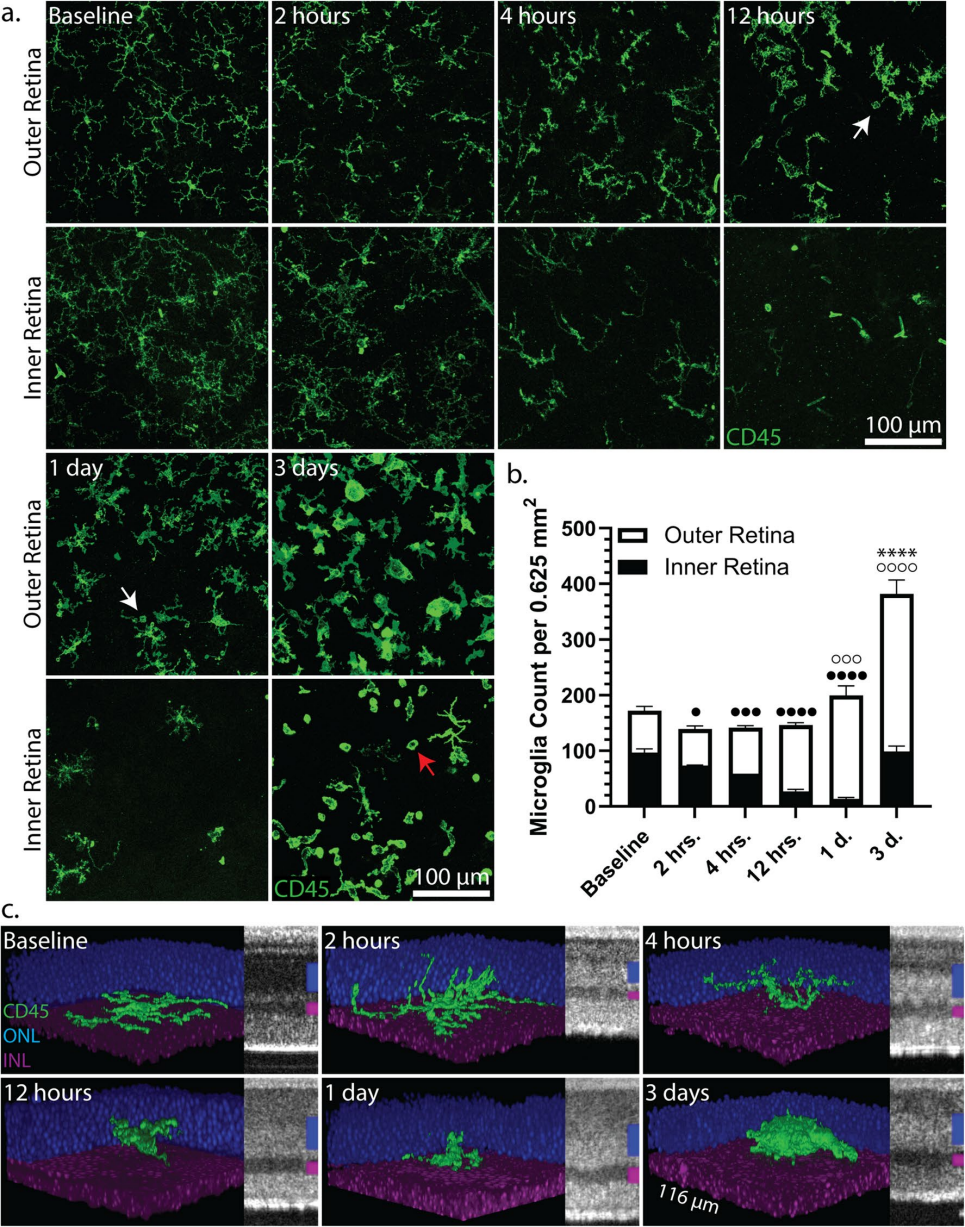
Microglia in the I307N *Rho* mouse migrate to the outer retina, change to an ameboid morphology, and expand in number after induction. I307N *Rho* mice were exposed to 20,000 lx of light for thirty minutes and eyes were enucleated before and between two hours and three days thereafter for preparation of PFA-fixed retinal flat-mounts and subsequent IF-staining for CD45 and DAPI. (**a**) Full-thickness images were captured in the area of light damage and Z-projections were created with ImageJ to depict CD45-positive cells (green) in the outer (OPL and ONL) or inner retina (IPL and GCL). Microglia responded by progressively changing from a ramified to an ameboid configuration. Isolated phagocytic events were observed at twelve hours and one day (white arrows). CD45-positive monocytic cells were seen in the inner retina on day three (red arrow). Original magnification = 10x. (**b**) Microglia were counted in the inner and outer retina in two separate images for a given animal and summed. The bar graph represents the mean count ± s.e.m. (*n* = 3 per time point). Microglia distribution changed as early as two hours after light damage. One-way ANOVA followed by Dunnett’s multiple comparisons tests was performed to compare the inner retina, outer retina, and total counts to baseline. Normality was tested with the Shapiro–Wilk test of residuals. Statistical symbols: black circle = inner retina; white circle = outer retina; * = total count. One-symbol = p < 0.05; Two-symbols = p < 0.01; Three-symbols = p < 0.001; Four-symbols = p < 0.0001. (**c**) 3D reconstructions (CD45 = green; ONL = blue; INL = purple) of isolated microglia (original magnification = 100x) at the interface of the OPL and ONL were created with ImageJ. Microglia dendrites rapidly infiltrated the ONL and began to retract as early as two hours and twelve hours, respectively. A frank ameboid morphology is achieved by day three. A 125 µm segment of the SD-OCT B-scan is provided to show the development of hyper-reflectivity alongside the microglia morphological changes (ONL: blue bar; INL: purple bar). The choroid and nerve-fiber layer (NFL) are positioned superiorly and inferiorly, respectively. Statistical data can be viewed in Supplementary Table S3.

Microglia migrated from the inner retina (IPL and GCL), beginning as early as two hours after light exposure, followed by repopulation of the inner retina on day three (Fig. 2a,b). When microglia at the interface of the OPL and ONL were manually isolated for 3D-reconstructions, dendrites could be seen infiltrating the ONL as early as two hours and four hours (Fig. 2c); individual phagocytic events were also apparent at high magnification at these time points (see Supplementary Fig. 2a). Continued phagocytosis from twelve hours through one day (Fig. 2a, white arrows) coincided with retraction of microglia dendrites until a fulminant ameboid morphology was attained by day three, likely after profound phagocytosis (Fig. 2c). Indeed, at the day three time point, a large fraction of the nuclei appeared pyknotic (Supplementary Fig. 2a). As time extended from day eight through one month after light exposure, microglia returned to a ramified morphology (see Supplementary Fig. 2b). Again, retinal microglia in wild-type littermates that were exposed to the same light damage protocol did not show evidence of morphological change (see Supplementary Fig. 2c). Furthermore, prominent co-localization of Iba1 and CD45 signal was observed in samples belonging to I307N *Rho* mice, offering further evidence that the CD45 signal was derived from microglia or macrophage (see Supplementary Fig. 2d).

**Figure 3:**
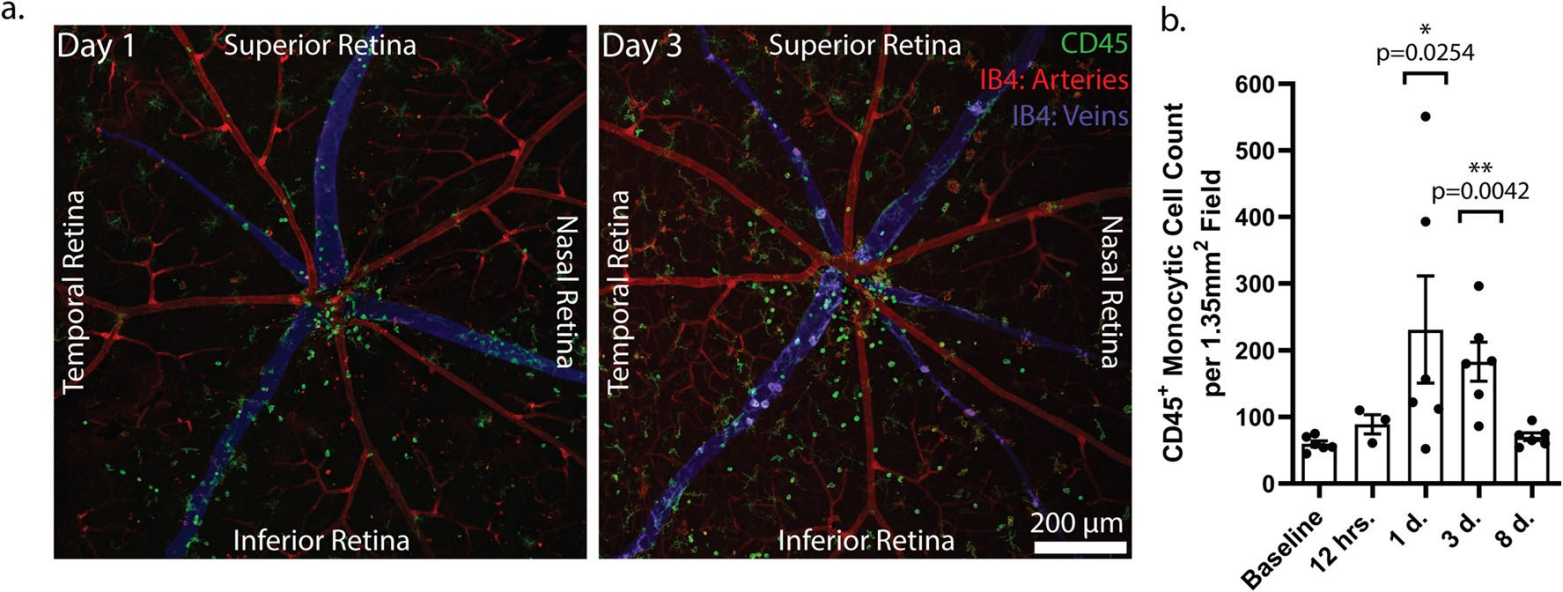
CD45-positive monocytic cells infiltrate the I307N *Rho* retina to a variable magnitude during retinal degeneration. PFA-fixed retinal flat-mounts were extracted from I307N *Rho* mice before or twelve hours, one day, three days, or eight days after light challenge and analyzed for CD45-positive monocytic cell infiltration. Full-thickness images were captured with an original magnification of 10x, representing a 1.34 mm^2^ area. Flat-mounts were stained for CD45-positive microglia (green) and IB4-positive retinal vasculature (red). (**a**) Representative Z-projections taken from days one and three after light challenge are shown. Pseudocolors were applied to depict the smaller diameter arteries with high IB4 binding (red) versus the larger diameter veins with low IB4 binding (blue). CD45-positive monocytic cells (green) were visualized infiltrating the ONH and veins on days one and three and appeared to migrate to areas between the major retinal veins on day three. (**b**) The total number of CD45-positive monocytic cells were counted through the entire thickness of each retinal flat-mount using the assistance of the 3D Objects Counter plugin^25^ in ImageJ. The highest mean counts for CD45-positive monocytic cells were seen on days one and three. The bar graph represents mean cell count  ±  *s.e.m*. (*n* = *6* per time point, excluding twelve hours when *n * = *3*). Statistical significance was determined with a non-parametric one-way ANOVA (Kruskal–Wallis test) followed by Dunn’s multiple comparisons tests that were performed to compare each time point to baseline. *p < 0.05; **p < 0.01.

CD45-positive monocytic cells were visualized within the inner retina, particularly on day three (Fig. 2a, red arrows). Since monocyte-derived macrophage infiltrate the retina of other mouse models of retinal degeneration^21–24^, we counted CD45-positive monocytic cells (Fig. 3a). The highest mean infiltration of CD45-positive monocytic cells occurred on days one and three post-light exposure when compared to baseline, though the magnitude of infiltration was highly variable (Fig. 3b). Indeed, the extent of CD45-positive monocytic cell infiltration likely reflects the severity of retinal degeneration, which can range from mild to severe even with the same dose of light^7^. We also observed that the CD45-positive monocytic cells appeared to infiltrate via the optic nerve head (ONH) and major retinal veins and noted that those in the day three samples appeared to be more dispersed when compared to day one (Fig. 3a).

### Microglia and Müller glia organize across the front of ONL hyper-reflectivity on SD-OCT

**Figure 4:**
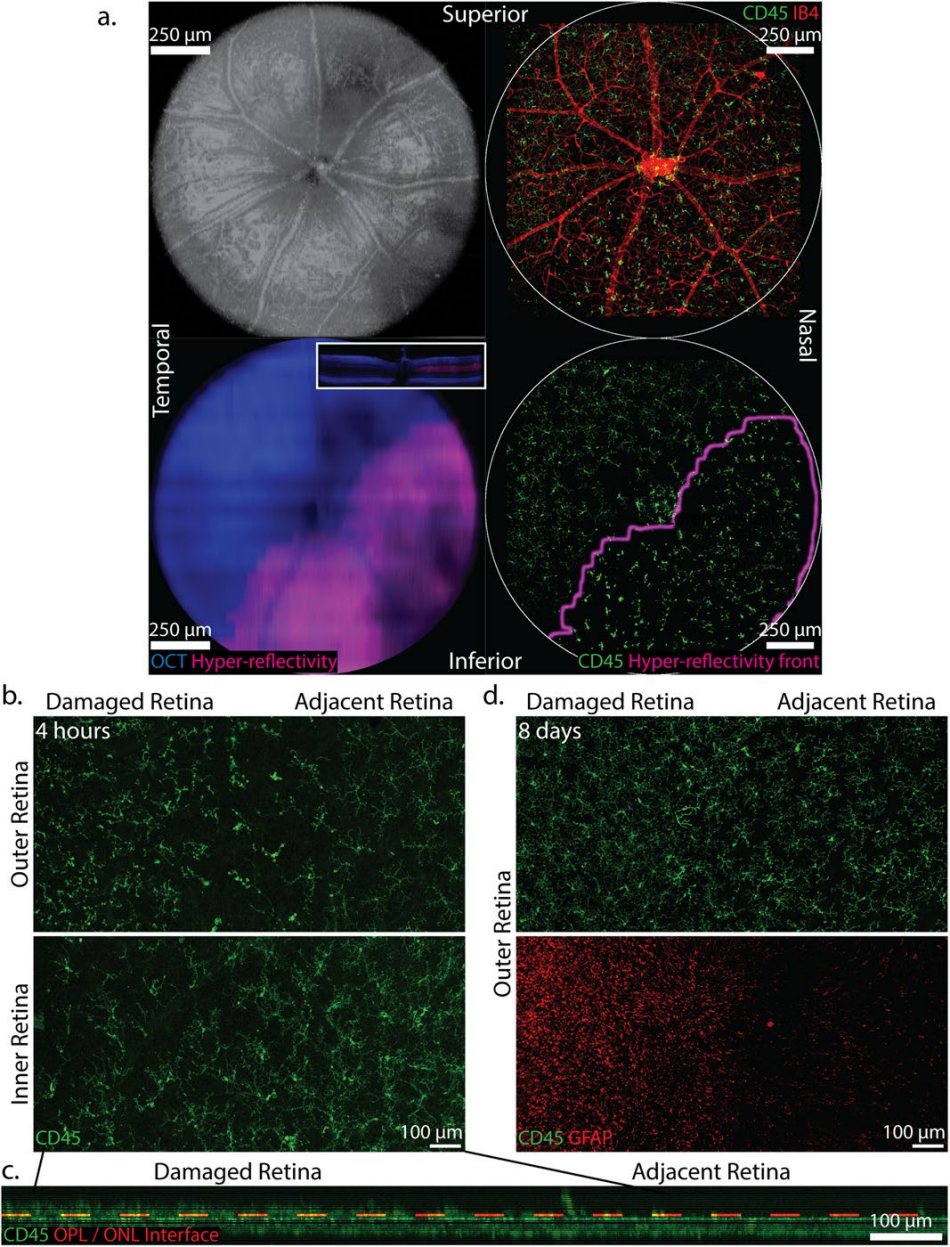
The SD-OCT hyper-reflectivity overlies the area of the retina that contains morphologically activated microglia. I307N *Rho* mice were challenged with thirty minutes of 20,000 lx of light and SD-OCT was performed at four hours (**a**–**c**) or eight days (**d**) after that followed by enucleation for IF-staining of PFA-fixed retinal flat-mounts. (**a**) *Top left panel:* an image of the fundus representing a 1.4 mm by 1.4 mm area of the retina was produced in ImageJ from SD-OCT B-scans. This enabled the retinal vasculature to serve as landmarks. *Top right panel:* an approximately 1.5 mm by 1.5 mm image of an IF-stained retinal flat-mount was captured with an original magnification of 7.5 × to depict the position and morphology of CD45-positive microglia (green) with reference to the IB4-positive retinal vasculature (red). The image was captured such that the vasculature in the fundus image and retinal flat-mount overlapped. Of note, the PFA-induced contraction and cover-slipping of the flat-mount prevented optimal alignment; therefore, the scale was carefully adjusted for improved alignment. *Bottom left:* the background SD-OCT and hyper-reflectivity signals from the top left panel were converted to blue and purple, respectively, using ImageJ. A Z-projection was then generated to depict the area of hyper-reflectivity in a fundus view. *Bottom right:* the front of hyper-reflectivity was isolated and merged with the CD45-signal (green) of the flat-mount using ImageJ, which demonstrated that morphologically distinct microglial populations were separated by the hyper-reflectivity front. (**b**) Morphologically distinct populations of microglia exist in close approximation in both the outer (ONL and OPL) and inner (IPL and GCL) retina at the four hour time point. Original magnification = 10x. (**c**) A cross-section of the CD45 signal (green) of the image in b. was produced with ImageJ. The dashed red line demarcates the interface of the OPL and ONL. CD45-positive microglia extended beyond this interface and thus infiltrated into the ONL in the damaged retina. (**d**) Morphologically distinct microglia (green) inhabited areas of the outer retina with differing levels of GFAP expression (red) on day eight. Original magnification = 10 ×.

We previously reported that a hyper-reflective signal develops in the ONL of the I307N *Rho* mouse during retinal degeneration on day one^7^. Therefore, we sought to determine if the area within which the microglia manifest an activated morphology coincides with the area of hyper-reflectivity on SD-OCT at the four hour time point when we both expected a mature hyper-reflective signal and distinct microglia morphology. To this end, we utilized the retinal vasculature as a reference to produce an overlay of a CD45 and Isolectin-B4 (IB4) co-stained retinal flat-mount and its corresponding SD-OCT hyper-reflective signal (Fig. 4a). Strikingly, morphologically distinct populations of microglia were observed across the hyper-reflectivity front in the XY-dimension (Fig. 4b), which was also seen in the Z-dimension as the microglia within the focus of light injury projected into the photoreceptor layer, beyond the interface of the OPL and ONL (Fig. 4c). The co-existence of morphologically distinct populations of microglia across the boundary of degeneration was evident at every time point up to one month (see Supplementary Fig. 3). Furthermore, CD45 and GFAP signal co-modulated across the front of injury on day eight when we expected intense microglia and Müller activation, suggestive of a coordinated response (Fig. 4d).

### Development of hyper-reflective SD-OCT signal, and transient retinal thinning, precedes the phase of profound microglial migration

**Figure 5:**
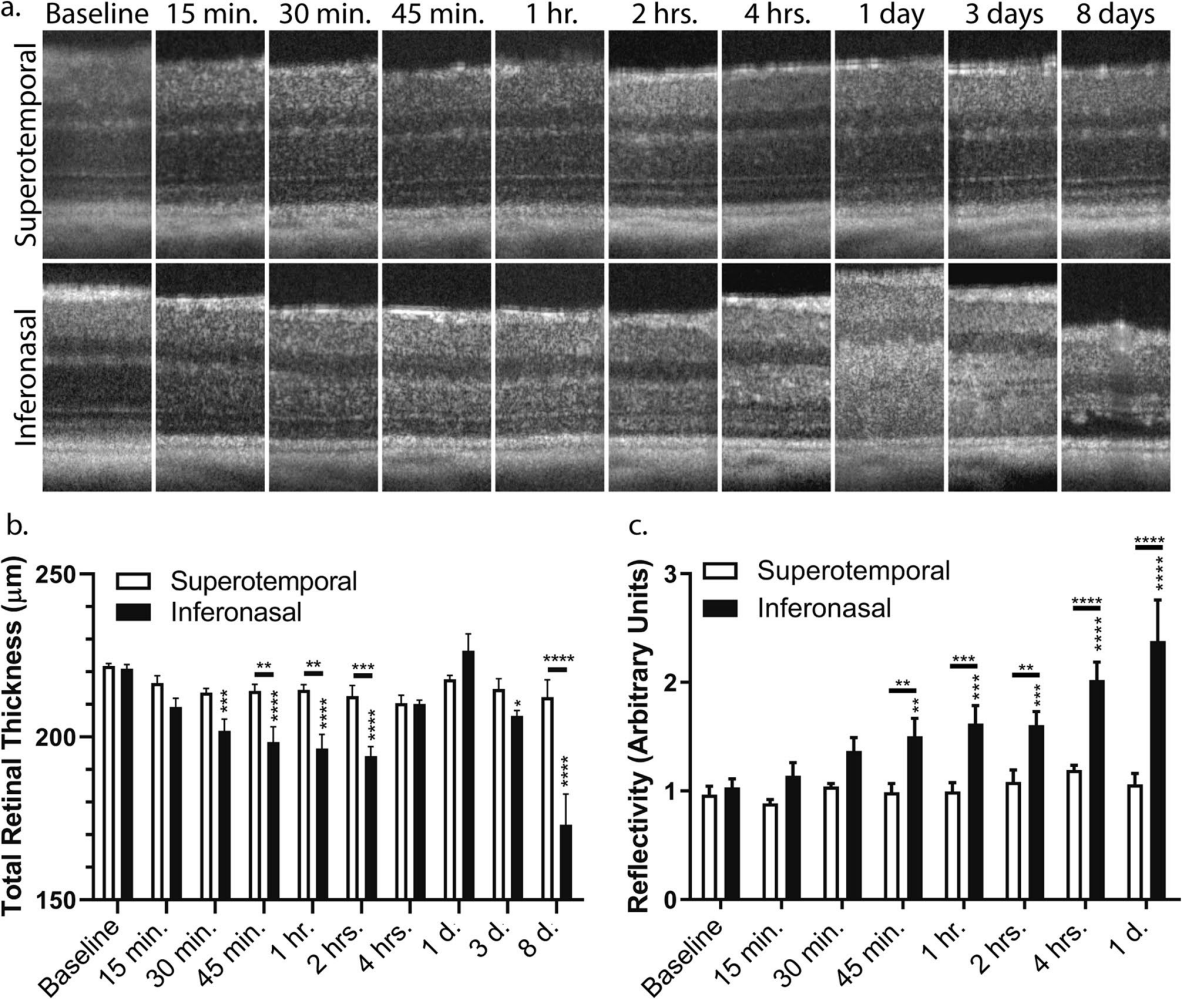
SD-OCT hyper-reflectivity and retinal thinning occur within one hour of exposure to bright white light. Four I307N *Rho* mice were challenged with 20,000 lx of light for thirty minutes. Serial SD-OCT scans were then obtained at multiple time points that spanned from fifteen minutes after the cessation of light exposure up to eight days thereafter. (**a**) Shown are representative 140 µm segments of SD-OCT B-scans from either the superotemporal or inferonasal retina at each time point for a single mouse. ONL hyper-reflectivity in the inferonasal retina was visually apparent as early as fifteen minutes and the retina appeared to thin after light treatment. (**b**) Total retinal thickness **(**TRT) was measured as the distance from the superficial aspect of the NFL to the deep aspect of the RPE. The TRT was recorded at six predetermined positions relative to the ONH, three in the superotemporal and inferonasal retina each, which were then averaged within and between eyes. (**c**) Longitudinal reflectivity profiles were obtained with ImageJ to measure reflectivity, which was recorded at the same three positions in the superotemporal and inferotemporal retinas as for the TRT and with the same averaging scheme. Measurements were not performed beyond the day one time point since we previously determined this to be the maximal response^7^. The bar graphs represent either the mean TRT or mean reflectivity ± *s.e.m.* (*n* = *4*). For both TRT and reflectivity, two-way repeated-measures ANOVA for matching data (for both time and retinal sector variables) followed by Tukey’s multiple comparisons tests were performed to determine statistical significance. The multiple comparisons tests compared each retinal segment at a given time point to its corresponding baseline (reported as asterisks above each bar graph) and to the opposite side of the retina at the same time point (reported as asterisks above each horizontal line segment). The factors of time and retinal sector, as well as their interaction, were statistically significant. Normality of residuals was assessed with Shapiro–Wilk test. *p < 0.05; **p < 0.01; ***p < 0.001; ****p < 0.0001. Statistical data can be viewed in Supplementary Table S4 and S5.

The development of a hyper-reflective signal in SD-OCT scans is a common feature of models of light-induced retinal degeneration in the acute phase of injury^26^. Interestingly, we observed that the hyper-reflective signal was well-developed by two hours after illumination when only microglial dendrites, and not cell bodies, had permeated the ONL (Fig. 2c), suggesting that the hyper-reflectivity was not derived from microglia in the ONL by themselves. To better address the contribution of microglia, if any, to the hyper-reflective signal, we performed serial SD-OCT scans of I307N *Rho* mice early after light exposure (Fig. 5a).

Unexpectedly, we observed that the retinal thickness decreased in the inferonasal retina soon after the cessation of light exposure. We thus measured the total retinal thickness (TRT) in the superotemporal and inferonasal retina where mild to no degeneration and maximal degeneration were expected, respectively. A significant reduction in TRT of the inferonasal retina was detected from thirty minutes to two hours after illumination. In support of our previous work^7^, TRT then increased from four hours to one day after light challenge before decreasing up to day eight (Fig. 5b). We measured ONL hyper-reflectivity in parallel with ImageJ (https://imagej.nih.gov/ij/) for each SD-OCT scan. We found that reflectivity appears as early as fifteen minutes after light exposure but achieves a statistically significant value only at forty-five minutes post illumination before continuing to increase up to day one (Fig. 5c). Of note, we previously demonstrated that the day one time point achieved the maximal hyper-reflective signal during a three day time course^7^. The magnitude of changes to TRT (both shrinking and swelling) was qualitatively proportional to the development of hyper-reflective signal (see Supplementary Fig. 4a). Importantly, wild-type littermates subjected to the same protocol did not exhibit significant changes in TRT or hyper-reflectivity (see Supplementary Fig. 4b). Taken together, the radical shifts in TRT may be the main contributor to producing the hyper-reflective signal rather than the infiltration of microglia of the ONL.

### Microglia migrate and transiently adhere to the RPE after light exposure

We previously showed that the RPE in the I307N *Rho* mouse remains morphologically and functionally intact fifteen days after light challenge despite rapid and robust degeneration of the ONL^7^. In their study, Zhang et al. employed microglia adherence to the RPE as an endpoint for microglial activation^11^. In addition, subretinal microglia may protect the RPE during light-induced retinal degeneration^27^. Given these observations, we sought to determine if microglial migration to the RPE is an early event and if microglia establish permanent residence at the RPE after light exposure.

**Figure 6:**
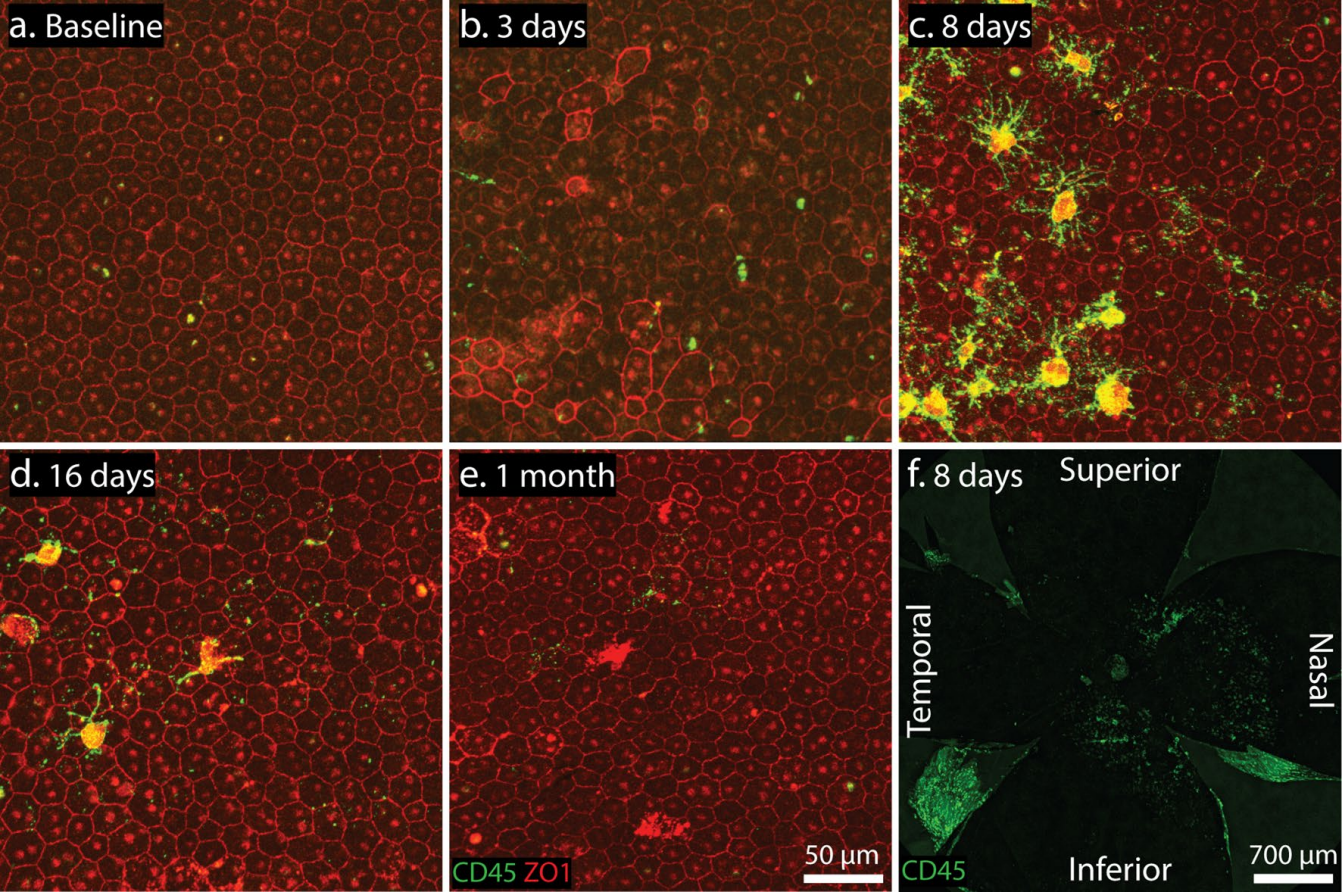
Microglia transiently adhere to the RPE during retinal degeneration in the I307N *Rho* mouse. PFA-fixed RPE flat-mounts were obtained from I307N *Rho* mice (**a**) before or (**b**) 3 days, (**c**) 8 days, (**d**) 16 days, or (**e**) 1 month after light challenge and stained for CD45-positive microglia (green) and the tight-junction protein ZO-1 (red), which is expressed at the RPE cell periphery. Uneven immunofluorescence with the ZO1 antibody may correspond to the puckering of the RPE monolayer. Obvious RPE dysmorphia was apparent on day three after the light treatment, which was before microglia established temporary residence on the RPE on days eight and sixteen. Autofluorescence, particularly in the 594 nm-channel, was observed at later time points. Original magnification = 20 ×. (**f**) A low-magnification image corresponding to the day eight condition was captured by merging consecutive images with an original magnification of 4 ×. Like microglia in retinal flat-mounts, microglia adhered to the RPE in an inferonasal pattern adjacent to the ONH.

We thus exposed I307N *Rho* mice to light and extracted their eyes on day three and up to one month after that for preparation of IF-staining of RPE flat-mounts with anti-CD45, anti-Iba1, or anti-zonula occludins-1 (ZO1), a junctional protein expressed at the periphery of RPE cells^28^ (Fig. 6). The day one time point was excluded since significant amounts of degenerated retina co-segregated with RPE during dissection. We observed that microglia established transient and late residence on the RPE, particularly on day eight (Fig. 6a–e). Microglial adherence to RPE also occurred in a sectoral pattern with an inferonasal bias (Fig. 6f). The co-localization of CD45 and Iba1 signal on RPE flat-mounts again demonstrated that these cells were likely microglia or macrophage (see Supplementary Fig. 5a). The RPE appeared grossly dysmorphic on day three after light exposure with both enlarged, irregular cells and small cells. Interestingly, dysmorphia existed predominantly along the edge of light damage (see Supplementary Fig. 5b–d). By day eight and afterward, the RPE resumed a more normal cobblestone appearance with isolated areas of irregular cells. These observations suggested that RPE partially recover histologically after an initial insult.

## Discussion

**Figure 7:**
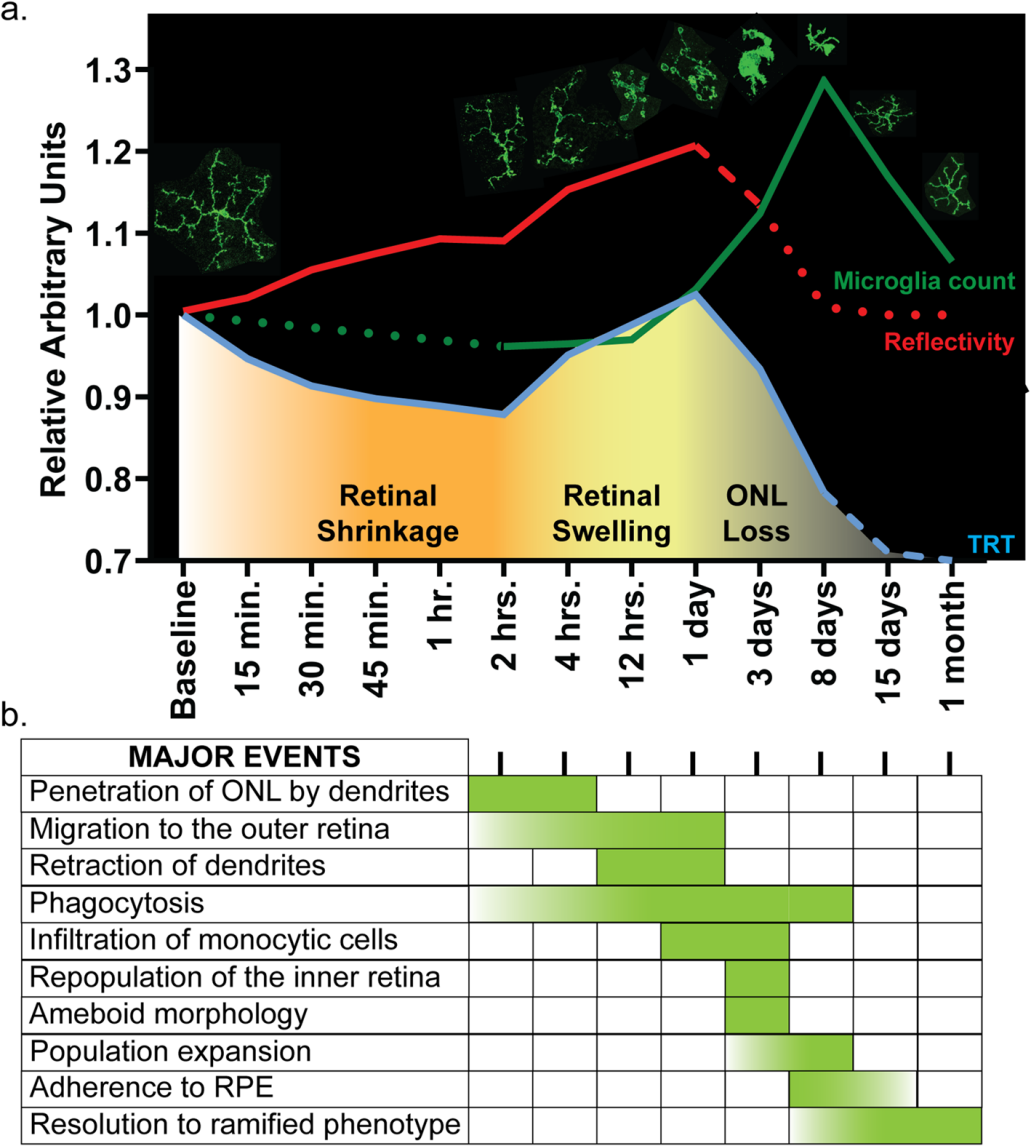
Summary figure correlating the major events of the microglial response with SD-OCT pathology during retinal degeneration in the I307N *Rho* mouse. (**a**) The graph depicts the mean values for total retinal thickness (TRT; blue), outer retina hyper-reflectivity (red), and the total microglia count relative to baseline across the entire time course. The solid line denotes that the curve is derived from data presented in Figs. 1 and 2 (green) or Fig. 4 (red and blue). The dashed curves are based on previous observations^7^ while the dotted curves represent predictions. The data used for the red and blue curves were transformed to fit within the range of the green curve. SD-OCT pathology can be organized into phases of retinal shrinkage, retinal swelling, and ONL loss, wherein all phases exhibit hyper-reflectivity. Micrographs of microglia in the outer retina demonstrate the typical morphology at each of the time points. (**b**) The major events of the microglia response are listed in the table provided and the shaded blocks (green) represent the presence of an event at one or multiple time points. When possible, a gradient was applied to the shaded area to indicate an increasing (white to green) or decreasing (green to white) effect. After light treatment, microglia (1) orient their dendrites towards photoreceptors, penetrate the ONL, and initiate phagocytosis as early as two hours and through four hours, (2) progressively migrate to the outer retina within two hours and through one day, (3) retract their dendrites and continue phagocytosis by twelve hours and through one day, (4) attain an ameboid morphology on day three and continue to phagocytose material until the ONL is cleared, (5) increase in number on days three through eight, (6) transiently adhere to the RPE on days eight through fifteen, and (7) return to a ramified phenotype beginning on day eight. Other important events include infiltration of the retina by CD45-positive monocytic cells on days one and three, and reconstitution of the inner retina population of microglia/macrophages on day three. These events predominantly overlap with retinal swelling and ONL loss phases on SD-OCT. The figure was created with Adobe Illustrator CC 2019 (www.adobe.com).

Our goal in utilizing the I307N *Rho* mouse has been to recapitulate the B1 phenotype of human adRP, which is characterized by the coexistence of areas of intense retinal degeneration with areas of healthy retina. Since many humans with the B1 phenotype experience more severe disease in the inferior and nasal retina, it has been proposed that asymmetric illumination from above (e.g. sunlight, room lighting) yields this sectoral pattern^9,17^. We previously demonstrated that exposure of the I307N *Rho* mouse to a bright overhead light induces retinal swelling, outer retina hyper-reflectivity, and eventual retinal thinning in SD-OCT scans with an inferonasal bias^7^. Our analysis here suggests that the inferonasal patterning is preserved at the histological level as a more profound increase in microglia and Müller glia reactivity occurred in the inferior retina. Moreover, the inferonasal hyper-reflectivity coincided with the region of the retina that contained morphologically active microglia. Our time course analysis further demonstrated the major events and timing involved in the response of microglia after light treatment**,** which can be correlated with the phases of retinal shrinkage, retinal swelling, and ONL loss on SD-OCT (Fig. 7).

Other research groups have induced the I307N *Rho* mouse with short durations of relatively low intensity, but diffuse, lighting in mirrored cages^6,8,10^. In our previous study, we found that longer durations and higher intensities were required to produce retinal degeneration when using overhead light^7^. The light damage protocol employed in the present study (30’ of 20,000 lx of light) was selected because it generated severe ONL loss, similar to that observed in the studies performed by *Budzynski* et al. and *Gargini* et al.^6, 8^. Given this, we expect that the results presented here regarding glial behavior in the I307N *Rho* mouse, specifically within the focus of light-induced retinal generation, are generalizable to other light damage protocols that are titrated to severe ONL loss. Indeed, microglia also achieved a fulminant ameboid morphology in the study by *Gargini* et al.^8^ However, since the position of the light source likely affects the area of the retina receiving illumination, differences in the spatial distribution of glial activation should be expected. To this point, *Zhang* et al. reported more TUNEL-positive photoreceptor nuclei in the superior retina after exposure of I307N *Rho* mice to light while in a white opaque polypropylene cage^11^.

The rate of retinal degeneration in humans and animal models of RP depends, in part, on the inciting gene defect^9,29^ and likely correlates with the magnitude of microglial morphological activation. Light-inducible models with swift retinal degeneration, including the I307N *Rho* and *arrestin*^−/−^ mice^26^, exhibit activated microglia with a qualitatively large size and a frank ameboid morphology, apparent on day three in our study. The *rd1* and *rd10* mouse models exhibit slower, but still rapid retinal degeneration that occurs in the weeks after eye opening and their microglia display smaller round bodies with few dendrites^30,31^. In the more slowly progressive P23H *RHO* rat and P23H *Rho* mouse, microglia retract their dendrites yet maintain an intermediate ramified phenotype^27,32,33^. Thus morphological activation of microglia towards a large, ameboid phenotype likely occurs in proportion to the size of the phagocytic bolus introduced by retinal degeneration, and may have important implications for cell signaling, such as the production of IL-1β^34^.

The boundary between damaged and preserved retina is a unique feature in the induced I307N *Rho* mouse and showed a steep gradient in microglia morphology, GFAP-expression by Müller glia, and partially reversible RPE injury. This organization points to potential, unique interactions among these cell types in close space^35^ that may not occur in models of global retinal degeneration that exhibit a more uniform distribution of microglia, like the P23H *RHO* rat^36^. Though we established correlation between Müller glia and microglia, we did not investigate the mechanisms of interaction between these cell types. However, crosstalk between Müller glia and microglia has been reported to help determine photoreceptor survival in other models^16,37–39^. Müller glia are capable of providing crucial neuroprotective support to photoreceptors, for example, by secreting LIF^40^, but can also contribute to the inflammatory response via secretion of cytokines^41,42^. Microglia similarly have shown evidence for neuroprotective and neurotoxic effects on photoreceptor survival^13,43^. Even more, subretinal microglia may protect the RPE during rapid light-induced retinal degeneration^27^. The seemingly opposing capabilities of microglia and Müller glia may appear incongruous during pan-retinal degeneration, but may be reconciled in the context of the lesion boundary in the I307N *Rho* mouse where pro-survival pathways and clearance of unsalvageable retina must co-exist in close but distinct spaces. Interestingly, *Stefanov* et al. noted that horizontal cell remodeling is most severe at the boundary of retinal degeneration in the I307N *Rho* mouse, which is suggestive of unique signaling events^10^.

Our data indicate that the number of microglia and/or macrophage increases after the infiltration of CD45-positive monocytic cells via the inner retinal veins and ONH. Our analysis, however, did not differentiate between endogenous microglia, infiltrating macrophage, and monocyte-derived macrophage^22–24,44–46^. Genetic techniques to differentiate microglia or monocyte-derived macrophage based on endogenous fluorescence have been employed by others^21,22^. These methodologies alongside single-cell RNA-seq have demonstrated that microglia and monocyte-derived macrophage may behave differently during retinal degeneration, both in terms of tropism within the retina, as endogenous microglia have displayed a bias towards populating the SRS in the P23H *Rho* knock-in mouse, and gene expression profiles^22,23,27^. Future studies with the I307N *Rho* mouse ought to examine the relative abundance of endogenous microglia versus monocyte-derived macrophage across the boundary of light-damage and assay for differences in phenotype^31^.

We found that microglia in the I307N *Rho* mouse reduce in number, move towards homeostatic positioning among the retinal layers, and exhibit a more ramified phenotype in the weeks following light exposure. Interestingly, microglia are capable of defaulting to the baseline, homeostatic transcriptome weeks after acute inflammation in endotoxin uveitis^47^. On the other hand, microglia in slowly progressive forms of RP may remain pro-inflammatory even after significant photoreceptor loss, which has been demonstrated in the P23H *RHO* rat^48^. Data from our present and past work with the I307N *Rho* model demonstrate that the ONL within the focus of light-induced retina can stably persist when mild retinal degeneration occurs^7^. Given this finding, determining whether microglia in the I307N *Rho* mouse can re-establish a homeostatic transcriptome after light exposure and what signals are required for such a transition in phenotype may be of value in illuminating novel therapeutic targets that could be exploited for the treatment of slowly progressive forms of RP.

As we suggested in our previous work, the origin of the outer retina hyper-reflectivity may illuminate potential therapeutic targets^7^. Though it is well-documented that retinal swelling occurs during light-induced retinal degeneration^26,49,50^, we found that TRT decreased when hyper-reflectivity initiated in the first hour after cessation of light exposure. Light adaptation in mice induces changes in retinal structures that are apparent via SD-OCT, including expansion of the SRS and photoreceptor outer segments, in part due to changes in osmotic forces and water movement during phototransduction^51–54^. Since overactivation of phototransduction may underlie retinal degeneration in the I307N *Rho* mouse^6^, the massive reduction in retinal thickness and hyper-reflectivity that occurs very early after light exposure could involve dysregulated water movement after a period of increased respiratory demand^54^ or G-protein-induced swelling of outer segments^53^. Fluid balance and retinal reflectivity may also be altered by Müller glia activity via modulation in the expression of aquaporin-4, the Kir4.1 potassium channel, or transient receptor potential isoform 4 (TRPV4) as well as changes to the integrity of the blood-retinal-barrier changes^49,55,56^. To this end, the I307N *Rho* mouse may help provide a link between novel pathological findings on SD-OCT, including the origin of retinal hyper-reflectivity, shrinking, and swelling, and associated mechanisms during retinal degeneration.

Our study presents an in-depth time course of the glial response during light-induced degeneration in the I307N *Rho* mouse and correlates these findings with SD-OCT imaging. Continued characterization of the I307N *Rho* mouse may uncover new insights regarding the B1 phenotype of adRP associated with mutations in *RHO* in human. Furthermore, comparison of the glial response after light-induction in the I307N *Rho* mouse and during retinal degeneration in animal models of slowly progressive and/or global RP could provide new perspectives for evaluating glial biology.

## Methods

### Animal models

All work abided by the Association for Research in Vision and Ophthalmology (ARVO) Statement for the Use of Animals in Ophthalmic and Vision Research and received prior approval from the Institutional Animal Care and Use Committee (IACUC) at the University of Florida. Animal breeding and genotyping was executed, as described in Massengill et al.^7^. Briefly, mice were reared in a 12:12-h dim-red light:dark lighting cycle with a maximum light intensity of 200 lx in the Animal Care Facility at the University of Florida. Genotyping via PCR was performed with forward and reverse primers with sequences of GGTCATCTTCTTCCTGATCTGCTGGC and TGCCAGCAGTCTGAGTGCAATG (IDT Technologies, Coralville, IA, USA), respectively, at a Tm of 61 ºC. The wild-type and I307N *Rho* PCR products (both ~ 260 bp products) were differentiated by restriction digestion with AflIII (New England Biosciences, Ipswich, MA, USA), where only the I307N *Rho* PCR product was cut and liberated two pieces of DNA with lengths of approximately 150 and 110 base-pairs, followed by agarose gel electrophoresis.

### Light damage protocol

A detailed protocol for light damage can be found in Massengill et al.^7^. Male and female mice were used for experimentation. Briefly, eight- to twelve-week old mice were dark adapted overnight in preparation for light damage. The following day, phenylephrine (Paragon BioTeck Inc., Portland, OR, USA) and atropine (Akorn, Lake Forest, IL, USA) were applied to the eye followed by a fifteen minute incubation, phenylephrine was then applied a second time followed by a second fifteen minute incubation, and phenylephrine was applied a third time immediately prior to challenging the mice with light. Ambulatory mice with maximally dilated eyes were placed into a cage with LED-lights fastened to its roof and the light was modulated to an intensity of 20,000 lx. Mice were exposed to the light for a thirty minute period and were utilized immediately for an experiment or returned to the 12:12-h dim-red light: dark lighting cycle. Light damage occurred between 4:00 PM and 12:00 AM.

### Spectral-domain optical coherence tomography (SD-OCT)

Eyes were dilated with phenylephrine (Paragon BioTeck Inc., Portland, OR, USA) approximately fifteen minutes prior to SD-OCT scanning. If a single scan was to be performed, mice were anesthetized with intraperitoneal injection of a cocktail containing 1 mg/mL of xylazine (Vedco, Saint Joseph, MO, USA) and 2.5 mg/mL of ketamine (Vedco, Saint Joseph, MO, USA) diluted in sterile saline (Vedco, Saint Joseph, MO, USA) at a dosage of 0.5 μL per gram of body mass. For time course experiments when more than one scan needed to be performed in a single day, mice were anesthetized with inhaled 2% isoflurane (Patterson Veterinary, Greeley, CO, USA) via a nose cone utilizing a SurgiVet Isotec vaporizer (Smiths Medical, Minneapolis, MN, USA).

Two SD-OCT scanning protocols were employed with the Envisu SD-OCT ophthalmic imaging system (Leica Microsystems, Durham, NC, USA). The first protocol was used for measurement of total retinal thickness (TRT) and reflectivity as well as production of figures with the retina in a cross-sectional view. Here, 25 B-scans representing the average of 10 individual frames were captured across a 1.4 mm × 1.4 mm window of the retina. The averaging ensured high image resolution in cross-section, but sacrificed resolution of the *en face* fundus view. The second protocol was used for generating figures with the retina in an *en face* fundus view and for determining the area encompassed by the front of hyper-reflectivity. Here, 200 B-scans were captured across a 1.4 mm × 1.4 mm window of the retina. This protocol allowed for high resolution of the *en face* fundus view at the expense of low resolution in cross-section. In both protocols, scans were done with the ONH at the center of the image. A discussion regarding the measurement of the TRT and reflectivity as well as image processing can be found in the Supplementary Information.

### Immunohistochemistry (IHC) of PFA-fixed frozen sections

Mice were euthanized by CO_2_ inhalation and cervical dislocation. Animal tattoo ink (Ketchum Mfg. Co., Lake Luzerne, NY, USA) was applied to the nasal portion of the cornea to maintain orientation. Eyes were enucleated, fixed with 4%-paraformaldehyde (PFA; Electron Microscopy Sciences, Hatfield, PA, USA) in PBS, dissected, cryo-protected in 30% sucrose, and frozen in Optimal Cutting Temperature Compound (Sakura, Torrance, CA, USA) as described in Massengill et al*.*^7^. Frozen OCT blocks were sectioned with a thickness of 16 μm along the superior to inferior axis at the level of the ONH with a Leica CM3050 S cryostat (Leica, Wetzlar, DEU) at − 20 °C. The Shandon Sequenza slide rack (Fisher Scientific, Hampton, NH, USA) was used for staining. Sections were hydrated with 500 μL PBS for ten minutes, permeabilized with PBS containing 0.5% Triton X-100 (PBS-T; Fisher Scientific, Hampton, NH, USA) with three 150 μL wash steps for five minutes each, incubated with 500 μL 0.5% NaBH_4_ (Sigma-Aldrich, St. Louis, MO, USA) in PBS three times for ten minutes each, washed with 500 μL PBS to remove the NaBH_4_, and incubated in 150 μL blocking buffer (10% normal horse serum in PBS-T; Vector Laboratories, Burlingame, CA, USA) for one hour at room-temperature. Primary antibody or antibodies were diluted in 150 μL of blocking buffer and incubated on the tissue at 4 °C overnight: rabbit anti-Iba1 1:500 (Wako, Osaka, JPN), goat anti-CD45 1:500 (R&D Systems, Minneapolis, MN, USA), and goat anti-GFAP 1:1000 (Abcam, Cambridge, UK). No primary antibody controls were incubated in blocking buffer only. Three 150 μL washes with PBS-T for five minutes each removed the primary antibody before application of 150 μL of secondary antibody or antibodies diluted to 1:2000 in blocking buffer: donkey anti-Goat IgG (H + L) Cross-Adsorbed Secondary Antibody Alexa Fluor 488 and donkey anti-Rabbit IgG (H + L) Secondary Antibody Alexa Fluor 594 (Thermo Fisher, Waltham, MA, USA). After a one hour incubation at room-temperature, secondary antibody solution was removed by washing three times with 150 μL of PBS-T for five minutes each as well as a single wash step with 500 μL of PBS. 500 μL of DAPI (Thermo Fisher, Waltham, MA, USA) diluted to 1:7000 in PBS was introduced to the slides for ten minutes followed by a final wash step with 500 μL of PBS. When staining for microglia (Iba1 or CD45), 150 μL of TrueBlack lipofuscin autofluorescence quencher (Biotum, Fremont, CA, USA) in 70% ethanol was applied to the slides for five minutes to minimize auto-fluorescence caused by oxidized lipid in highly phagocytic microglia. Two washes with 500 μL PBS removed the TrueBlack solution. Slides were cover-slipped with Fluoromount-G (Thermo Fisher, Waltham, MA, USA). A discussion regarding the measurement of the Müller glial and microglial response can be found in the Supplementary Information.

### IHC of retinal and RPE flat-mounts

Mice were euthanized by CO_2_ inhalation and cervical dislocation. Eyes were enucleated, fixed with 1%-PFA (Electron Microscopy Sciences, Hatfield, PA, USA) in PBS, dissected, and further fixed with 4%-PFA as described in Massengill et al*.*^7^. Tissues were incubated in 500 μL of PBS-T for thirty minutes at room-temperature for permeabilization. 500 μL of 0.5% Sodium Borohydride (Sigma-Aldrich, St. Louis, MO, USA) in PBS was applied to only the retina for thirty minutes at room-temperature then washed in PBS to reduce background staining. Retinal and RPE tissues were next incubated in 100 μL of blocking buffer (10% horse-serum in PBS-T; Vector Laboratories, Burlingame, CA, USA) overnight at 4 °C. Blocking buffer was removed and primary antibody or antibodies in 100 μL of blocking buffer were then applied to the tissues and incubated for one or three days for the RPE or retina, respectively: rabbit anti-Iba1 1:500 (Wako, Osaka, JPN), goat anti-CD45 1:500 (R&D Systems, Minneapolis, MN, USA), biotinylated GSL-IB4 from Griffonia simplicifolia 1:500 (Vector Laboratories, Burlingame, CA, USA), rabbit anti-GFAP 1:500 (Agilent Dako, Santa Clara, CA, USA), and rabbit anti-ZO1 1:200 (Invitrogen, Carlsbad, CA, USA). No primary antibody controls were incubated in blocking buffer only. Tissues were washed with 100 μL of PBS-T three times for five minutes each. Secondary antibody or antibodies diluted in 150 μL of blocking buffer were added to the tissue and incubated for one or two hours for RPE or retina, respectively: donkey anti-Goat IgG (H + L) Cross-Adsorbed Secondary Antibody Alexa Fluor 488 1:1000, donkey anti-Rabbit IgG (H + L) Secondary Antibody Alexa Fluor 594 1:000 (Thermo Fisher, Waltham, MA, USA), and Streptavidin Alexa Fluor 594 conjugate 1:500 (Invitrogen, Carlsbad, CA, USA). Tissues were washed with 100 μL of PBS-T three times for five minutes each, dissected into a clover configuration with four leaflets, flattened on a slide, and cover-slipped with Fluoromount-G (Thermo Fisher, Waltham, MA, USA). A discussion regarding image processing, cell counting, and the measurement of monocytic cell infiltration can be found in the Supplementary Information.

### Statistical analysis

Graphs were generated and statistical analyses were performed using GraphPad Prism Software, Version 8 (GraphPad Software Inc., San Diego, CA, USA). Normality of raw populations and residuals was assessed with the Shapiro–Wilk test. Two-way repeated measures ANOVA with matching for one factor (mixed-model ANOVA) followed by Sidak’s multiple comparisons test was employed to compare differences in retinal sector within the same animals, but differences across time between separate animals (Fig. 1c,d). Two-way repeated measures ANOVA with matching for both factors (retinal sector & time) followed by Tukey’s multiple comparisons test was used to compare differences in retinal sector across time within the same animal (Fig. 5b,c). Parametric one-way ANOVA with Dunnett’s multiple comparisons test (Fig. 2b) and non-parametric one-way ANOVA (Kruskal–Wallis test) with Dunn’s multiple comparisons test (Fig. 3b) was employed to compare differences in independent samples across time when normality was or was not met, respectively. An alpha level of 0.05 was employed.

## Supplementary information

Supplementary Information.

Supplementary Figure 1.

Supplementary Figure 2.

Supplementary Figure 3a.

Supplementary Figure 3b.

Supplementary Figure 4.

Supplementary Figure 5.

## Data Availability

The data produced in preparation of this manuscript are available at https://dataverse.harvard.edu/dataverse/Lewinlab.

## References

[1] Hamel CP (2014). Gene discovery and prevalence in inherited retinal dystrophies. C. R. Biol..

[2] Dias MF (2018). Molecular genetics and emerging therapies for retinitis pigmentosa: basic research and clinical perspectives. Prog. Retin. Eye Res..

[3] Daiger, S. P., Bowne, J. & Sullivan, L. S. *RetNet: Summaries of Genes and Loci Causing Retinal Diseases*, https://www.sph.uth.tmc.edu/RetNet/ (2018).

[4] Athanasiou D (2018). The molecular and cellular basis of rhodopsin retinitis pigmentosa reveals potential strategies for therapy. Prog. Retin. Eye Res..

[5] Ferrari S (2011). Retinitis pigmentosa: genes and disease mechanisms. Curr. Genom..

[6] Budzynski E (2010). Mutations of the opsin gene (Y102H and I307N) lead to light-induced degeneration of photoreceptors and constitutive activation of phototransduction in mice. J. Biol. Chem..

[7] Massengill MT (2018). Clinically relevant outcome measures for the I307N rhodopsin mouse: a model of inducible autosomal dominant retinitis pigmentosa. Invest. Ophthalmol Vis. Sci..

[8] Gargini C, Novelli E, Piano I, Biagioni M, Strettoi E (2017). Pattern of retinal morphological and functional decay in a light-inducible, rhodopsin mutant mouse. Sci. Rep..

[9] Cideciyan AV (1998). Disease sequence from mutant rhodopsin allele to rod and cone photoreceptor degeneration in man. Proc. Natl. Acad. Sci. USA.

[10] Stefanov A, Novelli E, Strettoi E (2020). Inner retinal preservation in the photoinducible I307N rhodopsin mutant mouse, a model of autosomal dominant retinitis pigmentosa. J. Comp. Neurol..

[11] Zhang X (2019). Wheel running exercise protects against retinal degeneration in the I307N rhodopsin mouse model of inducible autosomal dominant retinitis pigmentosa. Mol. Vis..

[12] Subirada PV (2018). A journey into the retina: Müller glia commanding survival and death. Eur. J. Neurosci..

[13] Silverman SM, Wong WT (2018). Microglia in the retina: roles in development, maturity, and disease. Annu. Rev. Vis. Sci..

[14] Campello L (2020). New Nrf2-inducer compound ITH12674 slows the progression of retinitis pigmentosa in the mouse model rd10. Cell Physiol. Biochem..

[15] Cammalleri M (2019). The urokinase-type plasminogen activator system as drug target in retinitis pigmentosa: new pre-clinical evidence in the rd10 mouse model. J. Cell Mol. Med..

[16] Roche SL, Ruiz-Lopez AM, Moloney JN, Byrne AM, Cotter TG (2018). Microglial-induced Müller cell gliosis is attenuated by progesterone in a mouse model of retinitis pigmentosa. Glia.

[17] Sumaroka A (2019). Autosomal dominant retinitis pigmentosa due to class B *Rhodopsin* mutations: an objective outcome for future treatment trials. Int. J. Mol. Sci..

[18] Tonks NK, Charbonneau H, Diltz CD, Fischer EH, Walsh KA (1988). Demonstration that the leukocyte common antigen CD45 is a protein tyrosine phosphatase. Biochemistry.

[19] Ekström P, Sanyal S, Narfström K, Chader GJ, van Veen T (1988). Accumulation of glial fibrillary acidic protein in Müller radial glia during retinal degeneration. Invest. Ophthalmol. Vis. Sci..

[20] Ito D (1998). Microglia-specific localisation of a novel calcium binding protein, Iba1. Brain Res. Mol. Brain Res..

[21] O'Koren EG, Mathew R, Saban DR (2016). Fate mapping reveals that microglia and recruited monocyte-derived macrophages are definitively distinguishable by phenotype in the retina. Sci. Rep..

[22] Ma W (2017). Monocyte infiltration and proliferation reestablish myeloid cell homeostasis in the mouse retina following retinal pigment epithelial cell injury. Sci. Rep..

[23] Ronning KE, Karlen SJ, Miller EB, Burns ME (2019). Molecular profiling of resident and infiltrating mononuclear phagocytes during rapid adult retinal degeneration using single-cell RNA sequencing. Sci. Rep..

[24] Karlen SJ (2018). Monocyte infiltration rather than microglia proliferation dominates the early immune response to rapid photoreceptor degeneration. J. Neuroinflamm..

[25] Bolte S, Cordelières FP (2006). A guided tour into subcellular colocalization analysis in light microscopy. J. Microsc..

[26] Levine ES (2014). Rapid light-induced activation of retinal microglia in mice lacking Arrestin-1. Vis. Res..

[27] O'Koren EG (2019). Microglial function is distinct in different anatomical locations during retinal homeostasis and degeneration. Immunity.

[28] Naylor A, Hopkins A, Hudson N, Campbell M (2019). Tight junctions of the outer blood retina barrier. Int. J. Mol. Sci..

[29] Collin GB (2020). Mouse models of inherited retinal degeneration with photoreceptor cell loss. Cells.

[30] Zhao L (2015). Microglial phagocytosis of living photoreceptors contributes to inherited retinal degeneration. EMBO Mol. Med..

[31] Zhou T (2017). Microglia polarization with M1/M2 phenotype changes in rd1 mouse model of retinal degeneration. Front. Neuroanat..

[32] Noailles A, Maneu V, Campello L, Lax P, Cuenca N (2018). Systemic inflammation induced by lipopolysaccharide aggravates inherited retinal dystrophy. Cell Death Dis..

[33] Viringipurampeer IA (2016). NLRP3 inflammasome activation drives bystander cone photoreceptor cell death in a P23H rhodopsin model of retinal degeneration. Hum. Mol. Genet..

[34] Fernández-Arjona MDM, Grondona JM, Fernández-Llebrez P, López-Ávalos MD (2019). Microglial morphometric parameters correlate with the expression level of IL-1β, and allow identifying different activated morphotypes. Front. Cell Neurosci..

[35] Natoli R (2017). Microglia-derived IL-1β promotes chemokine expression by Müller cells and RPE in focal retinal degeneration. Mol. Neurodegener..

[36] Noailles A, Fernández-Sánchez L, Lax P, Cuenca N (2014). Microglia activation in a model of retinal degeneration and TUDCA neuroprotective effects. J. Neuroinflamm..

[37] Hooper MJ, Ash JD (2018). Müller cell biological processes associated with leukemia inhibitory factor expression. Adv. Exp. Med. Biol..

[38] Di Pierdomenico J (2020). Coordinated intervention of microglial and müller cells in light-induced retinal degeneration. Invest. Ophthalmol. Vis. Sci..

[39] Arroba AI, Alvarez-Lindo N, van Rooijen N, de la Rosa EJ (2014). Microglia-Müller glia crosstalk in the rd10 mouse model of retinitis pigmentosa. Adv. Exp. Med. Biol..

[40] Ueki Y, Wang J, Chollangi S, Ash JD (2008). STAT3 activation in photoreceptors by leukemia inhibitory factor is associated with protection from light damage. J. Neurochem..

[41] Rutar M, Natoli R, Valter K, Provis JM (2011). Early focal expression of the chemokine Ccl2 by Müller cells during exposure to damage-inducing bright continuous light. Invest. Ophthalmol. Vis. Sci..

[42] Feng C (2017). Expression of CCL2 and its receptor in activation and migration of microglia and monocytes induced by photoreceptor apoptosis. Mol. Vis..

[43] Rashid K, Akhtar-Schaefer I, Langmann T (2019). Microglia in retinal degeneration. Front. Immunol..

[44] Zhang M, Xu G, Liu W, Ni Y, Zhou W (2012). Role of fractalkine/CX3CR1 interaction in light-induced photoreceptor degeneration through regulating retinal microglial activation and migration. PLoS ONE.

[45] Zhang Y (2018). Repopulating retinal microglia restore endogenous organization and function under CX3CL1-CX3CR1 regulation. Sci. Adv..

[46] Huang Y (2018). Dual extra-retinal origins of microglia in the model of retinal microglia repopulation. Cell Discov..

[47] Bell OH (2019). Single eye mRNA-Seq reveals normalisation of the retinal microglial transcriptome following acute inflammation. Front. Immunol..

[48] Noailles A (2016). Persistent inflammatory state after photoreceptor loss in an animal model of retinal degeneration. Sci. Rep..

[49] Geiger P, Barben M, Grimm C, Samardzija M (2015). Blue light-induced retinal lesions, intraretinal vascular leakage and edema formation in the all-cone mouse retina. Cell Death Dis..

[50] Aziz MK, Ni A, Esserman DA, Chavala SH (2014). Evidence of early ultrastructural photoreceptor abnormalities in light-induced retinal degeneration using spectral domain optical coherence tomography. Br. J. Ophthalmol..

[51] Li Y (2018). Light-dependent OCT structure changes in photoreceptor degenerative rd 10 mouse retina. Invest. Ophthalmol. Vis. Sci..

[52] Lu CD (2017). Photoreceptor layer thickness changes during dark adaptation observed with ultrahigh-resolution optical coherence tomography. Invest. Ophthalmol. Vis. Sci..

[53] Zhang P (2017). In vivo optophysiology reveals that G-protein activation triggers osmotic swelling and increased light scattering of rod photoreceptors. Proc. Natl. Acad. Sci. USA.

[54] Berkowitz BA (2018). Mitochondrial respiration in outer retina contributes to light-evoked increase in hydration in vivo. Invest. Ophthalmol. Vis. Sci..

[55] Iandiev I (2008). Localization of glial aquaporin-4 and Kir41 in the light-injured murine retina. Neurosci. Lett..

[56] Jo AO (2015). TRPV4 and AQP4 channels synergistically regulate cell volume and calcium homeostasis in retinal Müller glia. J. Neurosci..

