# Supplementary material for: Sectoral activation of glia in an inducible mouse model of autosomal dominant retinitis pigmentosa
Source: Sci Rep. 2020 Oct 12;10:16967. doi: 10.1038/s41598-020-73749-y (PMC7552392; doi:10.1038/s41598-020-73749-y)
Supplement: Supplementary file 1 — Supplementary Information. [file 41598_2020_73749_MOESM1_ESM.docx]

**Supplementary Information for:**

**Sectoral activation of glia in an inducible mouse model of autosomal dominant retinitis pigmentosa**

**Michael T. Massengill^1,^*, Neil F. Ash^1^, Brianna M. Young^2^, Cristhian J. Ildefonso^2^, Alfred S. Lewin^1,2^**

^1^ University of Florida College of Medicine, Department of Molecular Genetics and Microbiology, Gainesville, Florida, 32601, USA

^2^ University of Florida College of Medicine, Department of Ophthalmology, Gainesville, Florida, 32601, USA

* corresponding author: [*mmassen235@gmail.com*](mailto:mmassen235@gmail.com)

**Supplementary Methods:**

**SD-OCT: Total Retinal Thickness (TRT) and Reflectivity Measurements**

When taking the ONH at the center of SD-OCT scans as the origin of an XY coordinate system in the right eye (left = temporal, top = superior, right = nasal, inferior = temporal), TRT and reflectivity measurements were recorded at three positions in the superotemporal retina: (-233 µm, 466 µm), (-466 µm, 233 µm), (-233 µm, 233 µm), and three positions in the inferonasal retina: (233 µm, -466 µm), (466 µm, -233 µm), (233 µm, -233 µm). These coordinates were reflected across the vertical axis to analyze the left eye. TRT was measured as the distance from the superficial aspect of the NFL to the deep aspect of the RPE with the calipers in the Bioptigen Diver software (Leica Microsystems, Durham, NC, USA) at each of the above listed positions. For a given mouse, the three measurements in the superotemporal for each eye were averaged separately to obtain a within-eye average. The within-eye averages for the left and right eye were then averaged together to yield a final TRT measurement. The same algorithm was performed for the inferonasal retina.

A detailed protocol for the measurement of reflectivity with ImageJ is discussed in Massengill *et al.* ^7^. Briefly, longitudinal reflectivity profiles were generated at the same positions as listed for TRT using the Analyze Gel Function. The plots were subsequently reflected on the horizontal axis and a bounding box with a width of 75 pixels and height spanning the entire image was drawn to encompass the area corresponding to the outer nuclear layer (ONL) between the reflectivity peaks of the outer plexiform layer (OPL) and external limiting membrane (ELM). The area under the curve (AUC) was measured using the wand tool and reported as the reflectivity values. As with TRT, within eye averages followed by between eye averages for the superotemporal and inferonasal retina were performed.

**SD-OCT: *En face* Fundus Reconstruction and Producing the Hyper-reflectivity Front**

*En face* fundus reconstructions were produced in ImageJ ([*https://imagej.nih.gov/ij/*](https://imagej.nih.gov/ij/)). SD-OCT B-scans were exported as Tif files (200 per scan) and loaded as image sequences into ImageJ. The image scale was reset such that a pixel in the XY dimension was 1.4 µm and that a voxel in the Z dimension was 7 µm. The stack was cropped in the Y dimension to remove empty space above and below the retina and the Brightness/Contrast was adjusted to remove background noise. The stack was then re-sliced starting at the top and with an output spacing of 1.4 µm. The resliced stack was converted to an RGB Color Image-type, then converted to a temporal-color coded hyperstack with the Spectrum LUT, and then the color channels were split into red, green, and blue. The red and green channels were converted to an 8-bit image stack and a MAX-intensity Z-projection was generated. These image processing steps removed bright signal from the deep retinal layers (choroid and RPE predominantly) to allow for better detail and dimension of the retinal vasculature in the superficial layers.

The hyper-reflectivity front was produced with ImageJ using the same Tif files as described above for *en face* fundus reconstruction. Here, SUM-intensity Z-projections were created for groups of eight consecutive images such that the 200 image sequence was reduced to 25, each with higher cross-sectional resolution. An unedited version of the 25 image sequence was converted to blue to represent the background signal. A separate image sequence was converted to red and edited to isolate the hyper-reflective signal using the polygon selection tool and the clear outside function. The blue background and red hyper-reflectivity image sequences were merged, the image was re-sliced as described for the fundus reconstruction, and a MAX-intensity Z-projection was created to show the e*n face* hyper-reflective signal (now purple due to the red-blue overlay). To generate the outline of the hyper-reflectivity front, the red hyper-reflectivity MAX-intensity Z-projection was converted to an 8-bit image, a threshold was applied to create a binary signal, and an outline of the binary signal was applied. The outline was dilated, smoothed, and finally, a purple color was applied.

**IHC of PFA-Fixed Frozen Sections: Quantification of Müller Glia Reactivity and Microglia Number**

Four 40x fields of view of frozen cross-sections stained with GFAP and DAPI were imaged per eye with the Biorevo BZ-9000 microscope (Keyence, Osaka, JPN), two inferior to the ONH inside the area of visible damage and two superior to the ONH in a relatively unaffected area. The exposure times employed for imaging GFAP-expression were constant throughout the experiment. The GFAP-associated fluorescent channel of the resulting images was loaded into the ImageJ software for the determination of mean fluorescence intensity (MFI). The GFAP signal was measured as the MFI inside a polygon that was drawn to enclose the entire retina between the base of the photoreceptor inner segments and the nerve fiber layer (NFL). The MFI values obtained for the two inferior or two superior 40x fields for a given retina were averaged to generate a composite MFI in order to increase the robustness of the analysis. The relative MFI was calculated as the composite MFI for an area (inferior versus superior) of a given image divided by the average of the composite MFIs for the inferior images associated with the baseline condition.

Four 40x fields of view of frozen cross-sections stained with CD45, Iba1, and DAPI were imaged as described above. The CD45-associated fluorescent channel of the resulting images was loaded in the ImageJ software*.* Microglia were counted by two masked observers in each 40x field. CD45 signal was counted as a microglial cell if 1) a cell body was visualized, or 2) if multiple dendrites belonging to an individual cell appeared within frame. Small, highly fluorescent, and circular objects (monocytic cells) as well as isolated, single dendrites were not counted as microglia. The counts obtained from individual masked observers for the two inferior 40x fields were summed for a given retina, and the same was performed for the superior retina, in order to generate a composite count across a 725 µm retinal segment for individual observers. Counted microglia were then stratified based on their position by the masked observer (subretinal space, ONL, OPL, or inner retina). If a microglial cell spanned multiple layers, the layer that contained the majority of the cell was attributed to having contained that cell. Stratified counts were finally averaged between the individual masked observers. Interobserver agreement was assessed with the Pearson correlation in GraphPad Prism 8.0 (GraphPad Software Inc., San Diego, CA, USA).

**IHC of Retinal and RPE Flat-Mounts: Image Processing and Quantification of Microglia Number**

Full-thickness Z-stack images of retinal flat-mounts were obtained using the Leica DMi8 confocal microscope (Leica, Wetzlar, DEU) with either an original magnification of 10x, 20x or 100x. 10x and 20x images were utilized to demonstrate differences in CD45 and GFAP signal across the edge of injury (Figure 4, Supplementary Figure 3) and for *en face* views of microglia morphology (Figure 2, Supplementary Figure 2), respectively. In both cases, image sequences were divided into those belonging to the outer retina (OPL and ONL) or inner retina (IPL and GCL), and MAX-intensity Z-projections were generated with ImageJ; approximately half of the INL segregated with the inner and outer retina, each. Furthermore, the Brightness/Contrast was adjusted to enhance the signal to noise ratio and standardize the fluorescence intensity. In the case of the 20x views in Figure 2 and Supplementary Figure 2, images were cropped to 25% of their original size. 10x images across the lesion boundary were also used to create a cross-section to depict microglial distribution as a function of depth (Figure 4c, Supplementary Figure 3b). With ImageJ ([*https://imagej.nih.gov/ij/*](https://imagej.nih.gov/ij/)), the cross-section was generated using the 3D Project function after selecting the Brightest Point and X-axis of rotation settings. A dashed red line corresponding to the interface of the OPL and ONL was inserted by overlaying an image with red longitudinal bands at the appropriate slices within the full-thickness Z-stack images prior to initiating the 3D project function. Since images were co-stained with the IB4 retinal vasculature marker, the slices at the interface of the OPL and ONL was defined as the first two Z layers beyond the deep capillary plexus and into the ONL.

For cell counting in flat-mounts (Figure 2b), one MAX-intensity Z-projection was created in ImageJ for slices corresponding to the outer retina (ONL and OPL). Similarly, the layers corresponding to the inner retina (IPL and GCL) were compressed into four MAX-intensity Z-projections, which enabled simplification of counting. Approximately half of the INL was segregated with the outer and inner retina, each. Next, a five-pixel red dot was drawn over all CD45-positive cells, excluding intra- and extravascular monocytic cells, that appeared in these projections. The red dots were then counted by splitting the green (CD45) and red (dot) channels and using the 3D Objects Counter plugin ^31^ to calculate the number of binary objects within the split red channel.

100x images were utilized for 3D reconstructions of individual microglia that projected from the OPL into the ONL (Figure 2c). These images were also used to demonstrate nuclear pyknosis (Supplementary Figure 2a). Representative microglia were manually isolated using the polygon selection tool and the clear outside function in ImageJ. Pseudocolors were applied to the DAPI signal such that the ONL appeared blue and the INL appeared purple. Furthermore, 80% of the ONL was manually deleted so that the embedded microglia could be visualized after 3D rendering. As above, the Brightness/Contrast was adjusted to enhance the signal to noise ratio and standardize the fluorescence intensity. Stacks corresponding to microglia (green), the cropped ONL (blue), and INL (purple) were merged, and a 3D reconstruction was created using the ImageJ 3D viewer.

**IHC of Retinal and RPE Flat-Mounts: Quantification of Monocytic Cell Infiltration**

Full-thickness Z-stack images of retinal flat-mounts that were stained with anti-CD45 with or without IB4 were captured using the Leica DMi8 confocal microscope (Leica, Wetzlar, DEU) with the ONH situated at the center and an original magnification of 7.5x. Z-stacks were loaded into ImageJ for processing and analysis. Images corresponding to the avascular ONL were removed from each Z-stack since monocytes were unlikely to infiltrate this layer and the remaining frames were used to generate a MAX-intensity Z-projection of the CD45 signal to quantify the extent of CD45-positive monocytic cell infiltration. Next, a five-pixel red dot was drawn over all CD45-positive monocytic cells that appeared in the frame, including those that occupied an intravascular space; the original Z-stack was used as a reference if need. The red dots were then counted by splitting the green (CD45) and red (dot) channels and using the 3D Objects Counter plugin ^31^ to calculate the number of binary objects within the split red channel.

| ***Two-way ANOVA Summary*** | ***Interaction*** | ***Time*** | ***Sector*** | | ***Subject*** |
| --- | --- | --- | --- | --- | --- |
| P value | 0.0118 | <0.0001 | 0.0001 | | 0.2822 |
| P value summary | * | **** | *** | | ns |
| ***Dunnett's multiple comparisons test*** | | ***Summary*** | | ***Adjusted P Value*** | |
| ***Superior*** | | | | | |
| Baseline vs. Day 1 | | ns | | 0.0589 | |
| Baseline vs. Day 3 | | ns | | 0.9997 | |
| Baseline vs. Day 8 | | ns | | 0.1674 | |
| Baseline vs. Day 15 | | ns | | 0.6952 | |
| Baseline vs. Day 30 | | ns | | 0.9927 | |
|  | |  | |  | |
| ***Inferior*** | | | | | |
| Baseline vs. Day 1 | | ns | | 0.0589 | |
| Baseline vs. Day 3 | | * | | 0.0130 | |
| Baseline vs. Day 8 | | **** | | <0.0001 | |
| Baseline vs. Day 15 | | ** | | 0.0026 | |
| Baseline vs. Day 30 | | ns | | 0.8437 | |

**Supplementary Table S1: Statistical Analysis for Microglia Count in Cross-sections (Figure 1c)**

| ***Two-way ANOVA Summary*** | ***Interaction*** | ***Time*** | ***Sector*** | | ***Subject*** |
| --- | --- | --- | --- | --- | --- |
| P value | ns | * | ** | | ns |
| P value summary | 0.1201 | 0.0116 | 0.0019 | | 0.0894 |
| ***Dunnett's multiple comparisons test*** | | ***Summary*** | | ***Adjusted P Value*** | |
| ***Superior*** | | | | | |
| Baseline vs. Day 1 | | ns | | 0.7703 | |
| Baseline vs. Day 3 | | ns | | 0.4968 | |
| Baseline vs. Day 8 | | ns | | 0.1508 | |
| Baseline vs. Day 15 | | ns | | 0.2939 | |
| Baseline vs. Day 30 | | ns | | 0.9782 | |
|  | |  | |  | |
| ***Inferior*** | | | | | |
| Baseline vs. Day 1 | | ns | | 0.9996 | |
| Baseline vs. Day 3 | | ns | | 0.1572 | |
| Baseline vs. Day 8 | | ** | | 0.0023 | |
| Baseline vs. Day 15 | | ** | | 0.0039 | |
| Baseline vs. Day 30 | | ns | | 0.5192 | |

**Supplementary Table S2: Statistical Analysis for GFAP MFI in Cross-sections (Figure 1d)**

| ***One-way ANOVA Summary*** | ***Inner Retina*** | ***Outer Retina*** | ***Total*** |
| --- | --- | --- | --- |
| P value | <0.0001 | <0.0001 | <0.0001 |
| P value summary | **** | **** | **** |
| ***Dunnett's multiple comparisons test*** | | ***Summary*** | ***Adjusted P Value*** |
| ***Inner Retina*** | | | |
| Baseline vs. 2-hrs. | | * | 0.0294 |
| Baseline vs. 4-hrs. | | *** | 0.0009 |
| Baseline vs. 12-hrs. | | **** | <0.0001 |
| Baseline vs. 1-d. | | **** | <0.0001 |
| Baseline vs. 3-d. | | ns | 0.9985 |
|  | |  |  |
| ***Outer Retina*** | | | |
| Baseline vs. 2-hrs. | | ns | 0.9764 |
| Baseline vs. 4-hrs. | | ns | 0.9914 |
| Baseline vs. 12-hrs. | | ns | 0.1228 |
| Baseline vs. 1-d. | | *** | 0.0003 |
| Baseline vs. 3-d. | | **** | <0.0001 |
|  | |  |  |
| ***Total*** | | | |
| Baseline vs. 2-hrs. | | ns | 0.2647 |
| Baseline vs. 4-hrs. | | ns | 0.3311 |
| Baseline vs. 12-hrs. | | ns | 0.4627 |
| Baseline vs. 1-d. | | ns | 0.4192 |
| Baseline vs. 3-d. | | **** | <0.0001 |

**Supplementary Table S3: Statistical Analysis for Microglia Count in Flat-Mounts (Figure 2b)**

| ***Source of Variation*** | ***% of total*** | ***P value Summary*** | ***P value*** |
| --- | --- | --- | --- |
| Time | 38 | **** | <0.0001 |
| Sector | 17.64 | *** | 0.0002 |
| Time x Sector | 22.15 | **** | <0.0001 |
| ***Tukey's multiple comparisons test*** | | ***Summary*** | ***Adjusted P Value*** |
| Baseline:Superotemporal vs. 15 min:Superotemporal | | ns | 0.9816 |
| Baseline:Superotemporal vs. 30 min:Superotemporal | | ns | 0.6076 |
| Baseline:Superotemporal vs. 45 min:Superotemporal | | ns | 0.7187 |
| Baseline:Superotemporal vs. 1 hr:Superotemporal | | ns | 0.7703 |
| Baseline:Superotemporal vs. 2 hr:Superotemporal | | ns | 0.4178 |
| Baseline:Superotemporal vs. 4 hr:Superotemporal | | ns | 0.1363 |
| Baseline:Superotemporal vs. 1 d:Superotemporal | | ns | 0.999 |
| Baseline:Superotemporal vs. 3 d:Superotemporal | | ns | 0.8113 |
| Baseline:Superotemporal vs. 8 d:Superotemporal | | ns | 0.3572 |
|  | |  |  |
| Baseline:Inferonasal vs. 15 min:Inferonasal | | ns | 0.1058 |
| Baseline:Inferonasal vs. 30 min:Inferonasal | | *** | 0.0005 |
| Baseline:Inferonasal vs. 45 min:Inferonasal | | **** | <0.0001 |
| Baseline:Inferonasal vs. 1 hr:Inferonasal | | **** | <0.0001 |
| Baseline:Inferonasal vs. 2 hr:Inferonasal | | **** | <0.0001 |
| Baseline:Inferonasal vs. 4 hr:Inferonasal | | ns | 0.1864 |
| Baseline:Inferonasal vs. 1 d:Inferonasal | | ns | 0.9695 |
| Baseline:Inferonasal vs. 3 d:Inferonasal | | * | 0.0169 |
| Baseline:Inferonasal vs. 8 d:Inferonasal | | **** | <0.0001 |
|  | |  |  |
| 15 min:Superotemporal vs. 15 min:Inferonasal | | ns | 0.7632 |
| 30 min:Superotemporal vs. 30 min:Inferonasal | | ns | 0.1114 |
| 45 min:Superotemporal vs. 45 min:Inferonasal | | ** | 0.007 |
| 1 hr:Superotemporal vs. 1 hr:Inferonasal | | ** | 0.0012 |
| 2 hr:Superotemporal vs. 2 hr:Inferonasal | | *** | 0.0009 |
| 4 hr:Superotemporal vs. 4 hr:Inferonasal | | ns | >0.9999 |
| 1 d:Superotemporal vs. 1 d:Inferonasal | | ns | 0.5025 |
| 3 d:Superotemporal vs. 3 d:Inferonasal | | ns | 0.5994 |
| 8 d:Superotemporal vs. 8 d:Inferonasal | | **** | <0.0001 |

**Supplementary Table S4: Statistical Analysis for Total Retinal Thickness (TRT; Figure 5b)**

| ***Source of Variation*** | ***% of total*** | ***P value Summary*** | ***P value*** |
| --- | --- | --- | --- |
| Time | 25.13 | **** | <0.0001 |
| Sector | 34.07 | * | 0.014 |
| Time x Sector | 14.41 | **** | <0.0001 |
| ***Tukey's multiple comparisons test*** | | ***Summary*** | ***Adjusted P Value*** |
| Baseline:Superotemporal vs. 15 min:Superotemporal | | ns | >0.9999 |
| Baseline:Superotemporal vs. 30 min:Superotemporal | | ns | >0.9999 |
| Baseline:Superotemporal vs. 45 min:Superotemporal | | ns | >0.9999 |
| Baseline:Superotemporal vs. 1 hr:Superotemporal | | ns | >0.9999 |
| Baseline:Superotemporal vs. 2 hr:Superotemporal | | ns | 0.9968 |
| Baseline:Superotemporal vs. 4 hr:Superotemporal | | ns | 0.6565 |
| Baseline:Superotemporal vs. 1 d:Superotemporal | | ns | 0.9997 |
|  | |  |  |
| Baseline:Inferonasal vs. 15 min:Inferonasal | | ns | 0.9989 |
| Baseline:Inferonasal vs. 30 min:Inferonasal | | ns | 0.1382 |
| Baseline:Inferonasal vs. 45 min:Inferonasal | | ** | 0.0091 |
| Baseline:Inferonasal vs. 1 hr:Inferonasal | | *** | 0.0007 |
| Baseline:Inferonasal vs. 2 hr:Inferonasal | | *** | 0.001 |
| Baseline:Inferonasal vs. 4 hr:Inferonasal | | **** | <0.0001 |
| Baseline:Inferonasal vs. 1 d:Inferonasal | | **** | <0.0001 |
|  | |  |  |
| 15 min:Superotemporal vs. 15 min:Inferonasal | | ns | 0.4906 |
| 30 min:Superotemporal vs. 30 min:Inferonasal | | ns | 0.1604 |
| 45 min:Superotemporal vs. 45 min:Inferonasal | | ** | 0.0035 |
| 1 hr:Superotemporal vs. 1 hr:Inferonasal | | *** | 0.0003 |
| 2 hr:Superotemporal vs. 2 hr:Inferonasal | | ** | 0.003 |
| 4 hr:Superotemporal vs. 4 hr:Inferonasal | | **** | <0.0001 |
| 1 d:Superotemporal vs. 1 d:Inferonasal | | **** | <0.0001 |

**Supplementary Table S5: Statistical Analysis for Reflectivity (Figure 5c)**

**Supplementary Figure Legends:**

**Supplementary Figure 1. Controls for IF-stained cross-sections. a.** Representative images of IF-stained PFA-fixed frozen sections extracted from an I307N *Rho* mouse eight days after light exposure, demonstrating prominent co-localization of CD45 (green) and Iba1 (red) signal. This co-localization suggested that the CD45-positive cells are likely microglia or macrophage. **b.** Eyes from wild-type littermates were enucleated prior to or one day after exposure to thirty minutes of 20,000 lux of light for PFA-fixation, frozen sectioning, and subsequent IF-staining for CD45 and GFAP (both green), and DAPI (blue). Shown are representative images captured from the inferior retina. The dramatic morphological changes by microglia and upregulation of GFAP in Müller glia in the I307N *Rho* retinas that were exposed to bright white light are not evident in wild-type littermates. **c.** No primary antibody (Ab.) controls, incubated with blocking buffer containing 10% horse serum without primary antibody, for the day eight time point of an I307N *Rho* mouse when a robust microglial and Müller glial response would be expected. No microglia (CD45, Iba1) or Müller glia (GFAP) staining is observed. All images were captured with an original magnification of 40x.

**Supplementary Figure 2. Pyknotic nuclei appear within three days of light exposure and microglia activation resolves after clearance of the ONL. a.** Images taken within the ONL of retinal flat-mounts stained for CD45-positive microglia (green) and DAPI-positive nuclei (blue). Isolated phagocytic events were evident as early as two hours after exposure to light. Apparent pyknosis by DAPI staining developed by three days after light challenge. Most nuclei within ameboid microglia on day three were pyknotic, with only a few retaining their nucleolus (white arrow). Original magnification = 100x. **b.** Z-projections of retinal flat-mounts stained for CD45-positive microglia (green) within the damaged outer (OPL and ONL) or inner retina (IPL and GCL) on day eight through one month after challenging I307N *Rho* mice with bright white light. At these time points, microglia returned to a ramified morphology after undergoing dramatic morphological activation earlier after light treatment. **c.** Displayed are Z-projections of CD45-positive microglia (green) of IF-stained retinal flat-mounts belonging to wild-type littermates before or one day after exposure to light. Microglia in wild-type mice do not retract their dendrites as would be expected in the context of the degenerating I307N *Rho* retina at the one day time point. **d.** Z-projections of retinal flat-mounts that were extracted from an I307N *Rho* mouse three days after induction of retinal degeneration and stained for CD45-positive (green) and Iba1-positive (red) cells. There was prominent co-localization of the CD45 and Iba1 signals (composite), further suggesting that the CD45-positive cells were microglia or macrophage in origin. **e.** No primary antibody (Ab.) controls, incubated with blocking buffer containing 10% horse serum without primary antibody, for the day eight time point of an I307N *Rho* mouse when a robust microglia and Müller glia response would be expected. No staining of microglia (CD45, Iba1), Müller glia (GFAP), or vasculature (IB4) was observed. All images had an original magnification of 20x.

**Supplementary Figure 3. Morphologically distinct populations of microglia organize across the boundary between the damaged and adjacent, relatively undamaged retina.** I307N *Rho* mice were exposed to 20,000 lux of white light for thirty minutes and eyes were enucleated at multiple time points up to one month thereafter for preparation of PFA-fixed retinal flat-mounts and subsequent IF-staining for CD45 (green). **a & b.** Full-thickness images with an original magnification of 10x (1163 µm x 582 µm) were captured such that the damaged retina was situated on the left side of the frame and the adjacent, relatively undamaged retina to the right. Displayed are representative Z-projections of the outer retina (OPL and ONL) and inner retina (IPL and GCL) at each time point. Morphologically distinct microglia co-exist within a single frame across the X-dimension throughout the time course. **c.** Cross-sections of the images in a. up to the day three timepoint were produced with ImageJ. The dashed red line demarcates the interface of the OPL and ONL. CD45-positive microglia extended beyond this interface and thus infiltrated into the ONL in the damaged retina at each time point. The morphologically distinct microglia populations also exhibited differences in the Z dimension as microglia extended into the ONL of the damaged retina.

**Supplementary Figure 4. The magnitude of SD-OCT hyper-reflectivity and retinal thinning modulate with the extent of retinal injury in individual I307N *Rho* mice.** **a.** The line graph depicts the total retinal thickness (TRT) and reflectivity values for three individual mice from the experiment reported in Figure 5b & c, each mouse representing an increasing magnitude of injury from mild to severe. The top Y-axis segment and bottom Y-axis segment show the TRT and reflectivity, respectively, with increasing severity of injury from left to right. Furthermore, measurements are shown for the superotemporal (white circle) and inferonasal (black square) retina as separate lines. More profound fluctuations in retinal thinning and swelling, as well as higher reflectivity values, are achieved with increasing severity of injury. **b.** Two wild-type mice were similarly challenged with 20,000 lux of light for thirty minutes. Serial SD-OCT scans were obtained for each mouse at multiple time points that spanned from fifteen minutes after the cessation of light exposure up to eight days thereafter. The line graph again represents the TRT *(top Y-axis segment)* and reflectivity values *(bottom Y-axis segment)* for the individual wild-type mice in the superotemporal (white circle) and inferonasal (black square) retina. Significant changes in TRT and reflectivity were not observed in wild-type littermates that were exposed to bright white light.

**Supplementary Figure 5. CD45-positive cells that adhere to the RPE co-localize with Iba1 signal and RPE dysmorphia predominates at the edge of retinal degeneration.** Shown are representative images captured from PFA-fixed RPE flat-mounts, which were extracted from I307N *Rho* mice that had been subjected to the light damage protocol three or eight days prior. **a.** The sample was IF-stained for expression of CD45 (green; left panel) and Iba1 (red; middle panel) to assess for co-localization (composite; right panel). Given abundant overlapping of signal, the cells that adhered to the RPE were likely microglia or macrophage in origin. Original magnification of 40x. **b.** Representative RPE and retinal flat-mount, isolated on day three from the same eye of an individual animal. The RPE was stained with anti-ZO1 (red), which showed a focal, band-like nature of RPE dysmorphia. The retina was stained with CD45 (green) to demarcate the area of degenerating retina. The RPE dysmorphia co-localized with the edge of morphologically activated microglia. Original magnification = 4x. **c.** An image was captured with an original magnification of 10x across the injury boundary (approximate field of view shown as a white box in b.) to depict areas of relatively normal RPE in peripheral and central regions separated by significant RPE dysmorphia. **d.** The sample, isolated on day eight, was IF-stained for expression of ZO1 (red). The RPE attained an improved cobblestone appearance on day eight after an initial phase of dysmorphia on day three. Clumps of red-fluorescence co-localized with CD45-signal and likely represented autofluorescent material in highly phagocytic microglia (*data not shown*). Original magnification of 10x. **e.** The sample served as a no primary antibody (Ab.) control, which was incubated with blocking buffer containing 10% horse serum without primary antibody. A weak autofluorescence signal was visualized in both the green (488 nm) and red channels (594 nm), which was typical for later time points. However, the dendrites that were apparent in the anti-CD45 and anti-Iba1 treated samples are noticeably absent. Original magnification of 40x.
