# Supplementary figures and images for: Sectoral activation of glia in an inducible mouse model of autosomal dominant retinitis pigmentosa
Source: Sci Rep. 2020 Oct 12;10:16967. doi: 10.1038/s41598-020-73749-y (PMC7552392; doi:10.1038/s41598-020-73749-y)

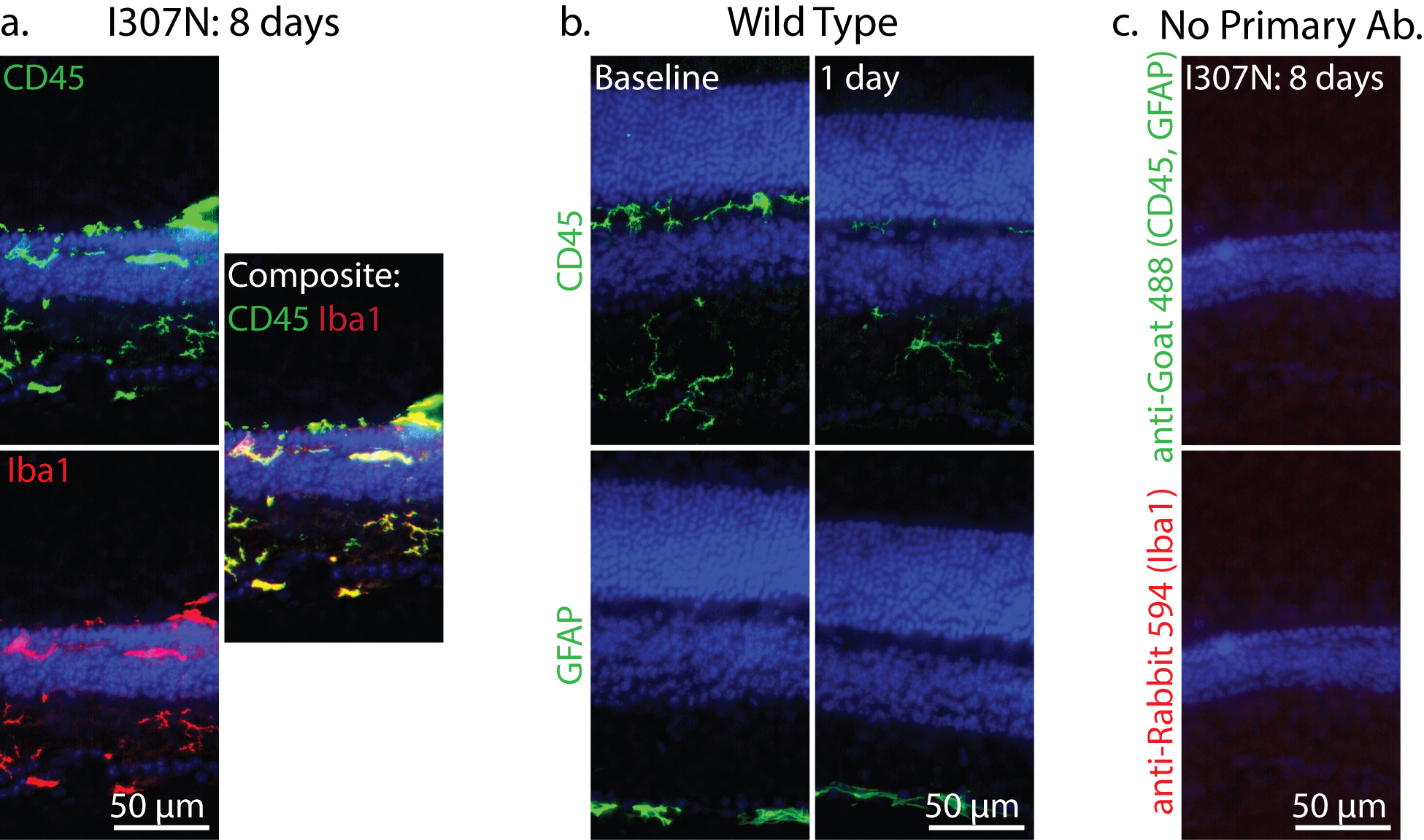

Supplement: Supplementary file 2 — Supplementary Figure 1. [file 41598_2020_73749_MOESM2_ESM.tif]

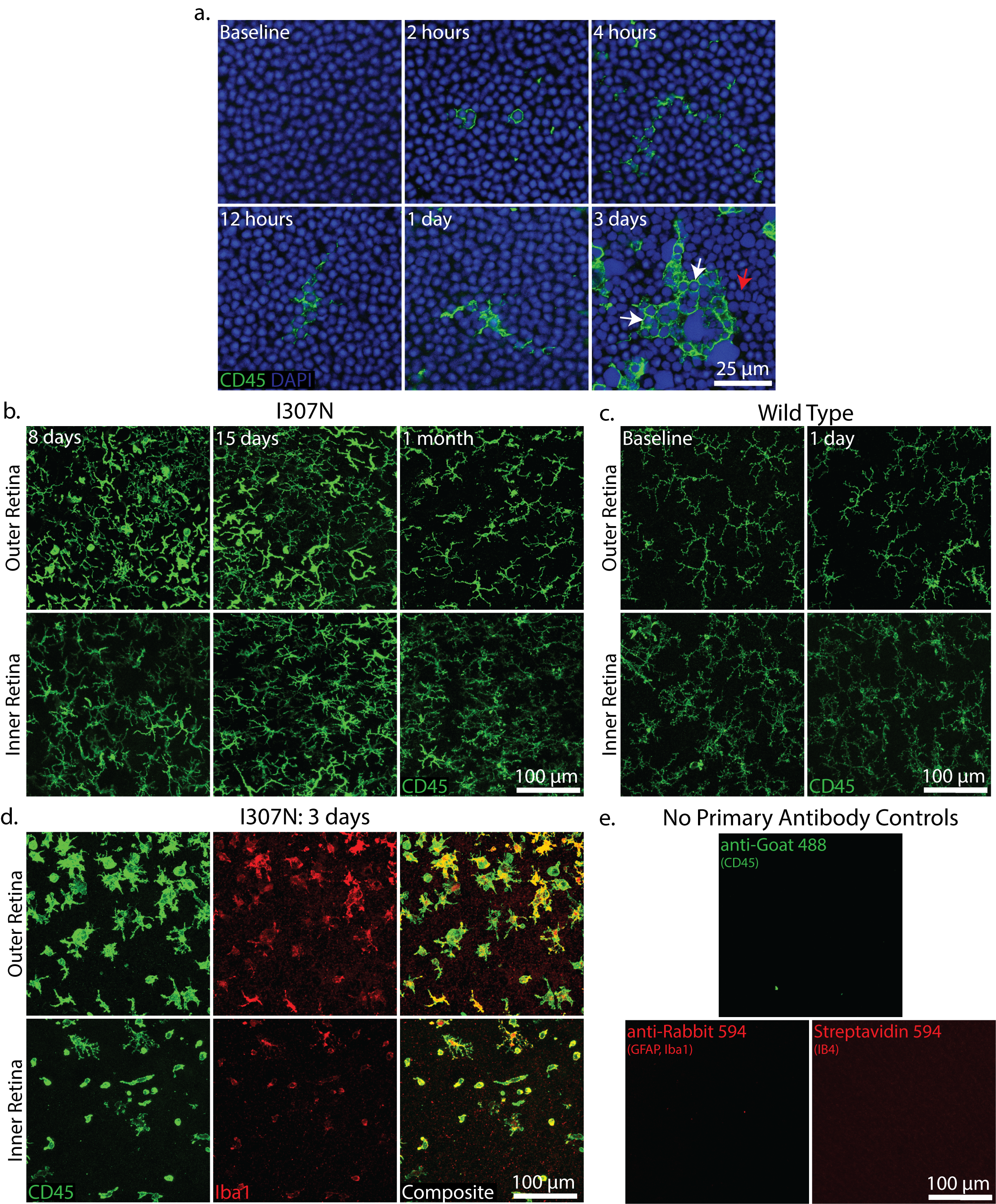

Supplement: Supplementary file 3 — Supplementary Figure 2. [file 41598_2020_73749_MOESM3_ESM.tif]

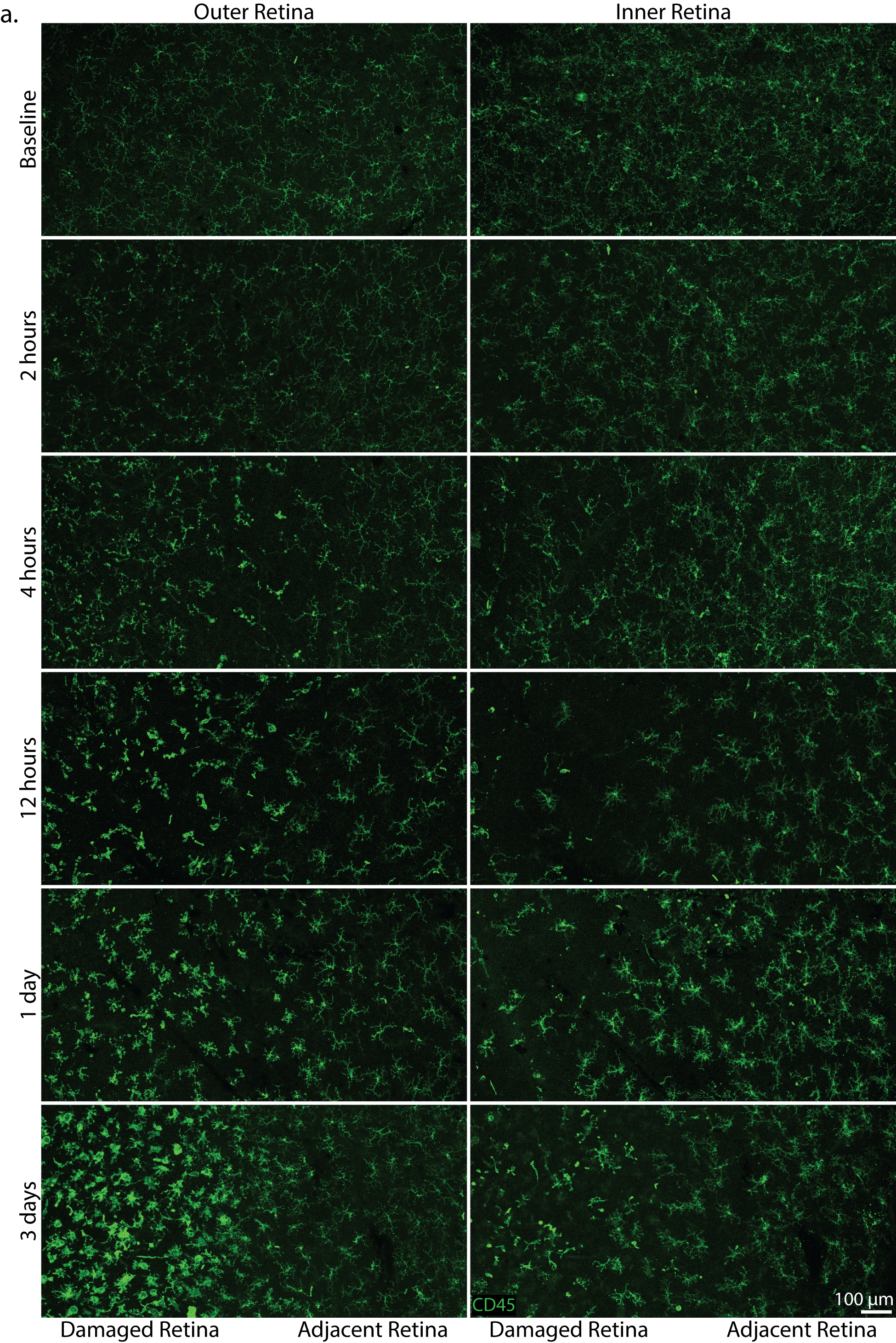

Supplement: Supplementary file 4 — Supplementary Figure 3a. [file 41598_2020_73749_MOESM4_ESM.tif]

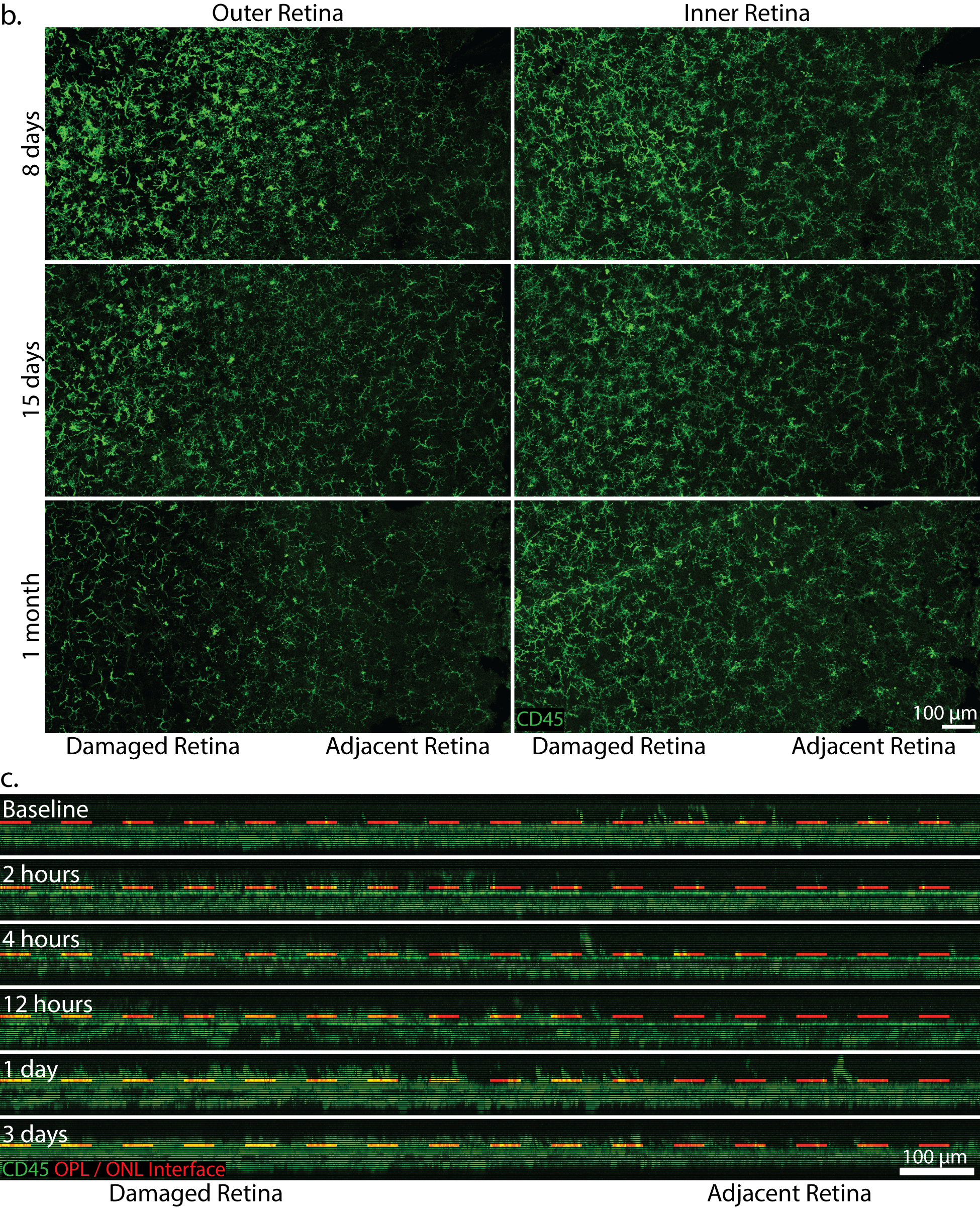

Supplement: Supplementary file 5 — Supplementary Figure 3b. [file 41598_2020_73749_MOESM5_ESM.tif]

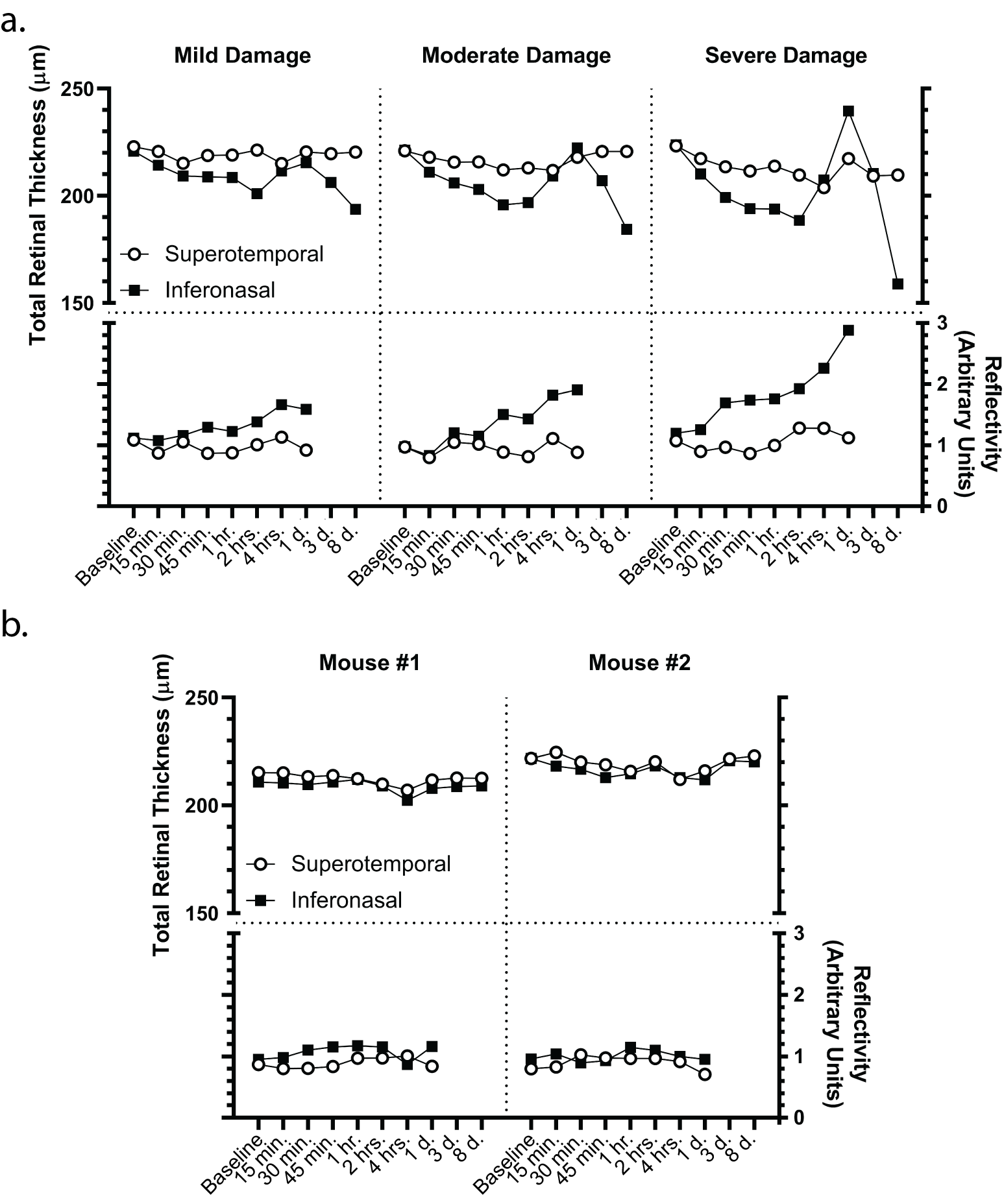

Supplement: Supplementary file 6 — Supplementary Figure 4. [file 41598_2020_73749_MOESM6_ESM.tif]

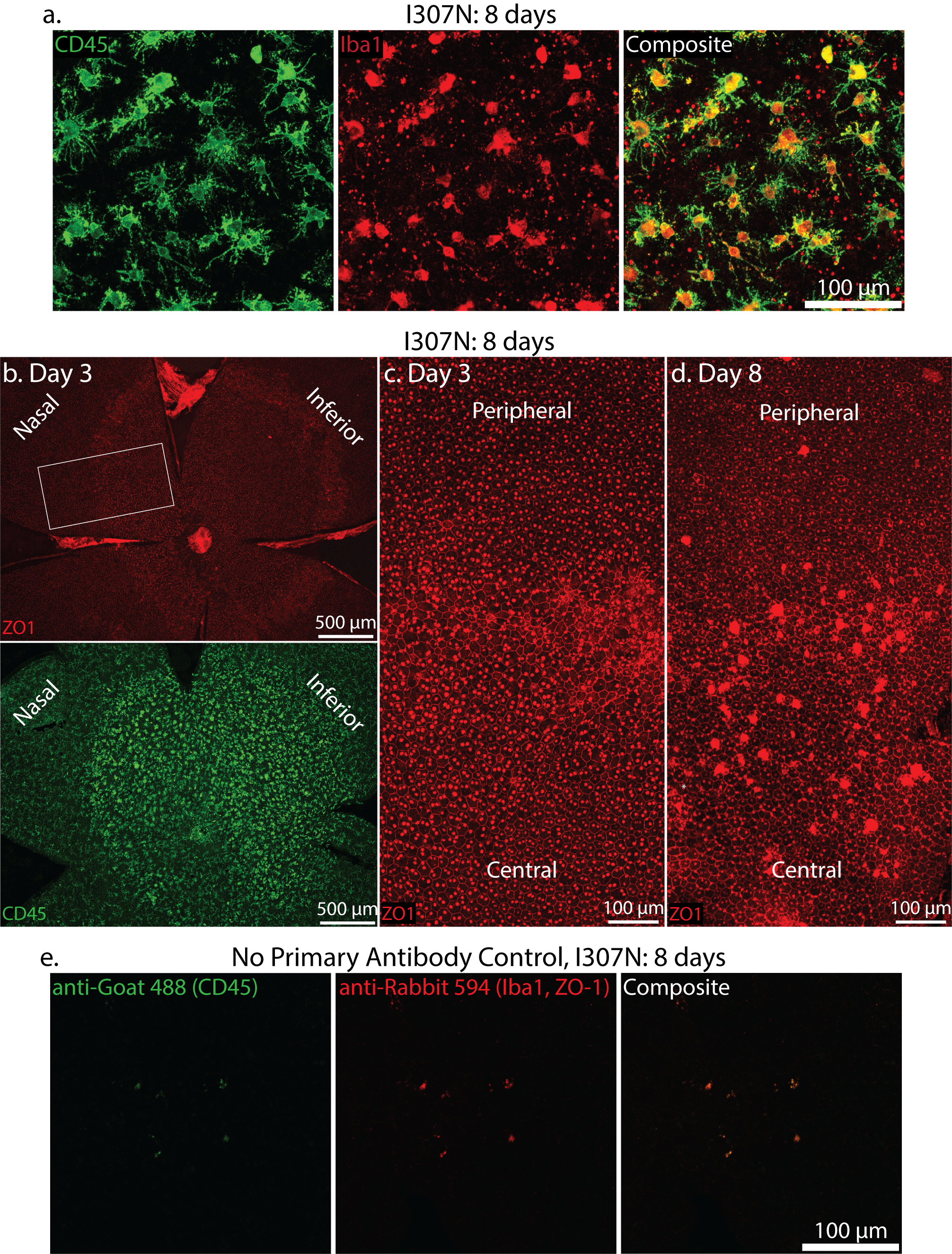

Supplement: Supplementary file 7 — Supplementary Figure 5. [file 41598_2020_73749_MOESM7_ESM.tif]
